# A DNA Damage-Induced, SOS-Independent Checkpoint Regulates Cell Division in *Caulobacter crescentus*

**DOI:** 10.1371/journal.pbio.1001977

**Published:** 2014-10-28

**Authors:** Joshua W. Modell, Tracy K. Kambara, Barrett S. Perchuk, Michael T. Laub

**Affiliations:** 1 Department of Biology, Massachusetts Institute of Technology, Cambridge, Massachusetts, United States of America; 2 Howard Hughes Medical Institute, Massachusetts Institute of Technology, Cambridge, Massachusetts, United States of America; CNRS, France

## Abstract

Cells must coordinate DNA replication with cell division, especially during episodes of DNA damage. The paradigm for cell division control following DNA damage in bacteria involves the SOS response where cleavage of the transcriptional repressor LexA induces a division inhibitor. However, in *Caulobacter crescentus*, cells lacking the primary SOS-regulated inhibitor, *sidA*, can often still delay division post-damage. Here we identify *didA*, a second cell division inhibitor that is induced by DNA damage, but in an SOS-independent manner. Together, DidA and SidA inhibit division, such that cells lacking both inhibitors divide prematurely following DNA damage, with lethal consequences. We show that DidA does not disrupt assembly of the division machinery and instead binds the essential division protein FtsN to block cytokinesis. Intriguingly, mutations in FtsW and FtsI, which drive the synthesis of septal cell wall material, can suppress the activity of both SidA and DidA, likely by causing the FtsW/I/N complex to hyperactively initiate cell division. Finally, we identify a transcription factor, DriD, that drives the SOS-independent transcription of *didA* following DNA damage.

## Abstract 2

A study of the bacterium *Caulobacter crescentus* reveals an SOS-independent DNA damage response pathway that acts via a novel cell division inhibitor, DidA, to suppress septum synthesis.

## Author Summary

Cells have evolved sophisticated mechanisms for repairing their DNA and maintaining genome integrity. A critical aspect of the repair process is an arrest of cell cycle progression, thereby ensuring that cell division is not attempted before the genome has been repaired and fully duplicated. Our paper explores the molecular mechanisms that underlie the inhibition of cell division following DNA damage in the bacterium *Caulobacter crescentus*. For most bacteria, the primary, and only mechanism previously described involves the SOS response, in which DNA damage induces cleavage of the transcriptional repressor LexA, driving induction of a battery of genes that includes an inhibitor of cell division (*sulA* in *E. coli* and *sidA* in *Caulobacter*). Here, we report that *Caulobacter* cells have a second, SOS-independent damage response pathway that induces another division inhibitor, *didA*, which works together with *sidA* to block cell division following DNA damage. We also identify the damage-sensitive transcription factor responsible for inducing DidA. Finally, our study demonstrates that DidA and SidA inhibit cell division in an atypical manner. Many division inhibitors in bacteria appear to inhibit the protein FtsZ, which forms a ring at the site of cell division. DidA and SidA, however, target a trio of proteins, FtsW/I/N, that help synthesize the new cell wall that will separate the daughter cells (the septum). In sum, our work expands our understanding of how bacterial cells respond to DNA damage and the mechanisms by which they regulate cell division.

## Introduction

Progress through the cell cycle requires the sequential execution of three fundamental processes: DNA replication, chromosome segregation, and cell division. Maintaining the precise order of these events is crucial to preserving genomic integrity, as any attempt to divide before completing DNA replication or chromosome segregation could result in the scission of DNA and a failure to endow each daughter cell with a complete genome. Coordinating DNA replication and cell division is particularly challenging when cells encounter DNA damaging agents that necessitate lengthy periods of chromosome repair. To ensure the order of cell cycle events and preserve genome integrity, many cells employ checkpoints that actively halt cell cycle progression until DNA damage has been repaired. While checkpoints are prevalent and well characterized in eukaryotes [1], their role and significance in governing the bacterial cell cycle is less clear.

The α-proteobacterium *C. crescentus* is an excellent system for understanding the bacterial cell cycle. Cells are easily synchronized and DNA replication initiates once and only once per cell division, resulting in distinguishable G1, S, and G2 phases. As with most bacteria, cell division in *Caulobacter* involves the assembly of a large multiprotein complex at mid-cell that drives constriction of the cell envelope and separation of daughter cells [2]. The position of the division machinery, known as the “divisome,” is established by the tubulin homolog FtsZ, which forms a ring-like structure at mid-cell and subsequently recruits other essential cell division proteins [2]–[4]. Once assembled, how these proteins coordinate the various steps of cytokinesis is unclear and the factor(s) that ultimately trigger cytokinesis are unknown.

Like eukaryotes, bacteria can inhibit cell division following DNA damage. The best studied mechanism involves the “SOS response” [5],[6] in which DNA damage stimulates the recombinase RecA to trigger an autocatalytic cleavage of the transcriptional repressor LexA. This cleavage leads to induction of SOS genes, many of which are involved in DNA recombination and repair [6],[7]. The SOS regulon also typically includes a cell division inhibitor that can delay cytokinesis until after damage is cleared. The best characterized SOS-induced division inhibitor, *Escherichia coli* SulA, disrupts polymerization of FtsZ and thus inhibits assembly of the divisome [8],[9]. However, *sulA* is not widely conserved beyond the γ-proteobacteria and recent studies have indicated that the SOS-induced division inhibitors from several Gram-positive species do not target FtsZ, although in most cases the direct target remains unknown [10]–[12].

In *Caulobacter* the primary SOS-induced division inhibitor is a 29 amino acid inner membrane protein called SidA that inhibits division by interacting with the late-arriving division protein FtsW [13]. Although *sidA* is the primary SOS-induced division inhibitor in *Caulobacter*, cells lacking *sidA* can still arrest division when grown in the presence of the DNA damaging agent mitomycin C (MMC). An SOS-regulated endonuclease called BapE may indirectly contribute to inhibiting division [14], but we conjectured that *Caulobacter* encodes another direct cell division inhibitor that is induced by DNA damage but in an SOS-independent manner. Here, we identify such an inhibitor, now named *didA*. As with *sidA*, the overexpression of *didA* in undamaged cells is sufficient to prevent cell division. Cells lacking both inhibitors divide prematurely following DNA damage, leading to a significant viability defect. DidA does not disrupt FtsZ ring formation or divisome assembly and instead likely inhibits division through an interaction with the divisome component FtsN. Intriguingly, point mutations in FtsW and FtsI, which help drive septal cell wall synthesis, suppress the lethality that results from overproducing either SidA or DidA. Our results suggest that these mutations hyperactivate the cell division process and implicate the protein complex FtsW/I/N in the triggering of cytokinesis. Finally, we identify a transcription factor, DriD, that activates *didA* expression, thus revealing the basis of a damage-inducible, but SOS-independent pathway in *Caulobacter*.

## Results

### Identification of *didA*, a DNA Damage-Induced, SOS-Independent Cell Division Inhibitor

Our previous work demonstrated that s*idA* is the primary SOS-induced division inhibitor in *Caulobacter*. However, many Δ*sidA* and Δ*recA* cells exposed to the DNA damaging agent MMC still become filamentous suggesting that an SOS-independent inhibitor may also prevent division following DNA damage (Figure S1) [13]. To identify candidate inhibitors, we examined global gene expression changes following MMC treatment of a *ΔrecA* strain, which cannot induce SOS genes. Wild-type and *ΔrecA* cells were grown to mid-exponential phase in rich medium and exposed to MMC for 30 minutes. RNA was then isolated and compared to mock treated cells on whole genome DNA microarrays (Data S1A).

**Figure 1:**
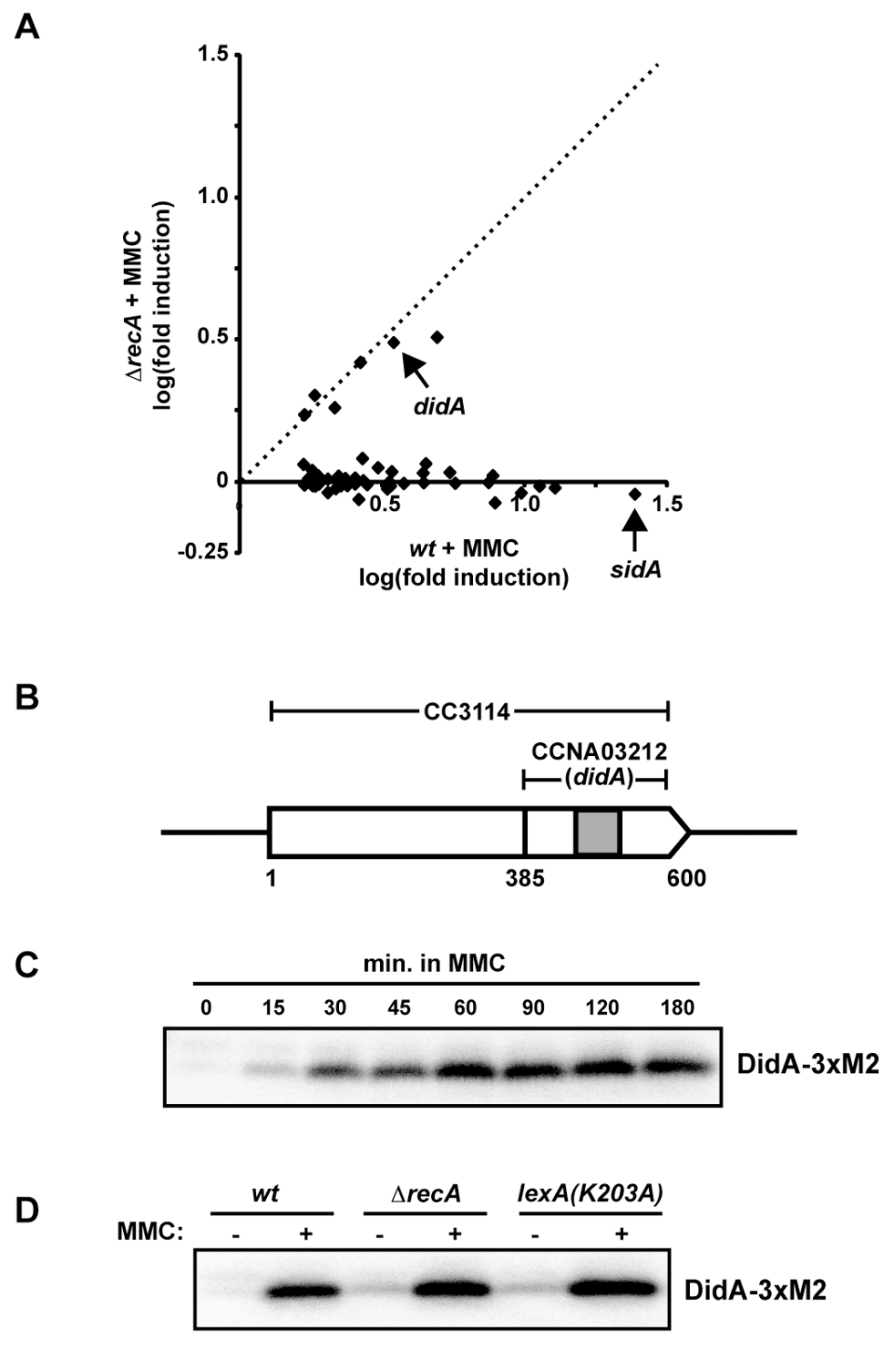
*didA* is induced by DNA damage and is not SOS regulated. (A) Wild-type and Δ*recA* cells were grown in rich medium to mid-exponential phase and treated with 1 µg/ml MMC for 30 minutes. Expression values, the average of two biological replicates, are shown for the 50 most upregulated genes in wild-type cells with fold-change ratios calculated in comparison to mock treated cells. The dashed line corresponds to fold-change values that are identical in wild-type and *ΔrecA* cells. For complete data, see Figure S2 and Data S1A. (B) CC3114 and CCNA03212 (*didA*) are shown schematically in their genomic context. Nucleotide positions relative to the annotated CC3114 start site are shown below. The gray shaded region represents a predicted transmembrane domain. (C) Western blot of cells producing DidA fused to a C-terminal 3×M2 epitope from the chromosomal *didA* locus. Cells were grown to mid-exponential phase and treated with 1 µg/ml MMC for the times indicated. (D) Western blot of wild-type, Δ*recA* and *lexA(K203A)* cells expressing *didA-3×M2* from its native locus treated with 1 µg/ml MMC for 1 hour. Membranes (C–D) were blotted with the α-FLAG/M2 antibody.

Of the 50 most upregulated genes following MMC treatment in wild-type cells, 44 were *recA*-dependent, including 31 that are directly regulated by LexA (Figure 1A and S2A) [13],[15]. The remaining six damage-regulated genes showed similar induction levels in both wild-type and *ΔrecA* backgrounds (Figure 1A) and are thus likely controlled by an SOS-independent mechanism. One of these genes, CCNA03212 in the NA1000 (CB15N) genome, encodes a previously uncharacterized 71 amino acid protein with a single predicted transmembrane helix flanked by short cytoplasmic and periplasmic domains (Figure 1B). The open reading frame of CCNA03212 overlaps with the C-terminus of the open reading frame of CC3114, annotated in the closely related strain CB15. In our expression profiling experiments, only those probes lying within the CCNA03212 coding sequence were significantly upregulated in wild-type cells treated with MMC (Figure S2B and S2C), suggesting that the NA1000 annotation is correct. Based on the studies described below, we named this gene *didA* (for damage-induced cell division inhibitor A).

To confirm that *didA* encodes a damage-inducible protein, we created a strain in which the chromosomal *didA* gene was fused to the coding region of the 3×M2 epitope. This C-terminal fusion, DidA-3×M2, was barely detectable in the absence of DNA damage, but was strongly induced following MMC treatment with protein levels increasing nearly 20-fold after 1 hour (Figure 1C). Western blotting indicated a band at the size predicted for DidA-3×M2 (∼11 kDa) and not CC3114-3×M2 (∼25 kDa) indicating that the larger gene product annotated in CB15 is not produced at significant levels in these conditions. To test the SOS-dependence of DidA-3×M2 synthesis following MMC treatment, we examined DidA-3×M2 production in a Δ*recA* strain and in a strain harboring *lexA(K203A)*, which encodes a noncleavable form of LexA that blocks the induction of SOS genes. In each case, DidA-3×M2 was slightly elevated in untreated cells, likely due to increased basal levels of damage in the absence of SOS-mediated repair (Figure 1D). Following MMC treatment, DidA-3×M2 was strongly induced in all strains (Figure 1D), consistent with an SOS-independent mode of regulation.

**Figure 2:**
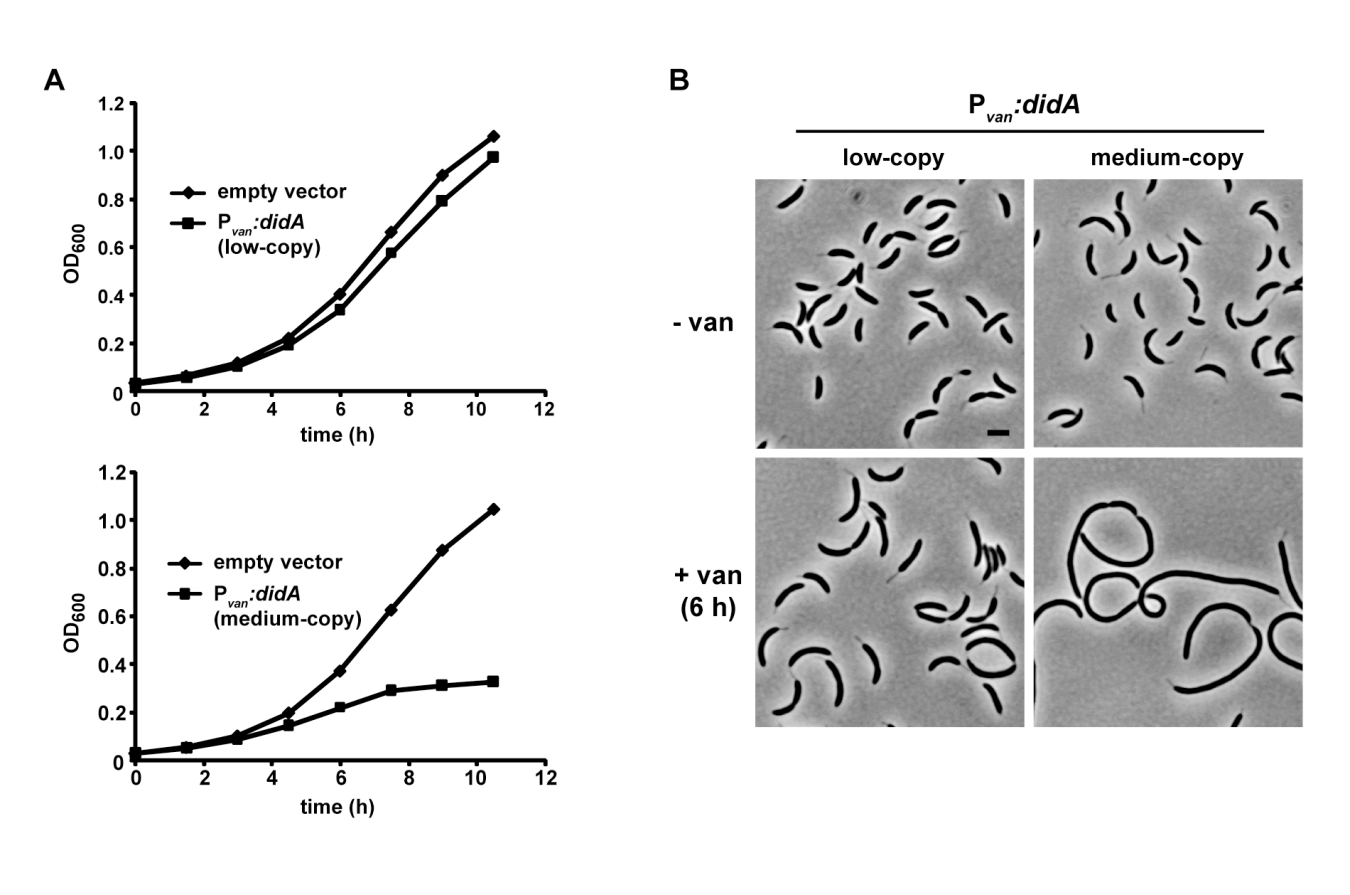
DidA is sufficient to inhibit cell division. Growth curves (A) and micrographs (B) of strains overexpressing *didA*. Cells harboring a low- or medium-copy plasmid that expresses *didA* from the vanillate-inducible promoter P*_van_* were grown in rich medium with or without vanillate for the times indicated. Bar, 2 µm.

To test whether DidA can inhibit cell division, we fused the *didA* coding sequence to the vanillate-inducible promoter P*_van_* and cloned this construct into both low- and medium-copy plasmids. We transformed wild-type cells with each plasmid and then grew cells in the presence of vanillate to induce *didA* in the absence of a DNA damaging agent. Synthesis of DidA from the low-copy plasmid resulted in mild cellular filamentation and a modest growth defect, while overproduction from the medium-copy plasmid caused a more pronounced division defect with nearly all cells demonstrating severe filamentation after 6 hours (Figure 2A and 2B). Thus, DidA, like SidA, is sufficient to inhibit cell division in the absence of DNA damage.

To assess the level of DidA accumulation during our overproduction experiments, we fused the coding region for a 3×M2 tag to the 5′ end of *didA* and expressed this construct from its native promoter on the chromosome or from the P*_van_* promoter on a low- or medium-copy plasmid. After 3 hours of induction, cells producing DidA from either plasmid became filamentous indicating that 3×M2-DidA is functional (Figure S3A). As expected, cells expressing *3×M2-didA* from the native chromosomal locus also became filamentous following treatment with MMC. Importantly, the levels of 3×M2-DidA that led to filamentation when produced from either plasmid were slightly lower than that seen when produced from the native locus during MMC exposure (Figure S3B), indicating that the phenotypes observed in Figure 2 are not the result of artificially high DidA levels. Taken together, our results suggest that following DNA damage, DidA accumulates in an SOS-independent fashion to help prevent cell division.

### SidA and DidA Redundantly Regulate Division during MMC Treatment

**Figure 3:**
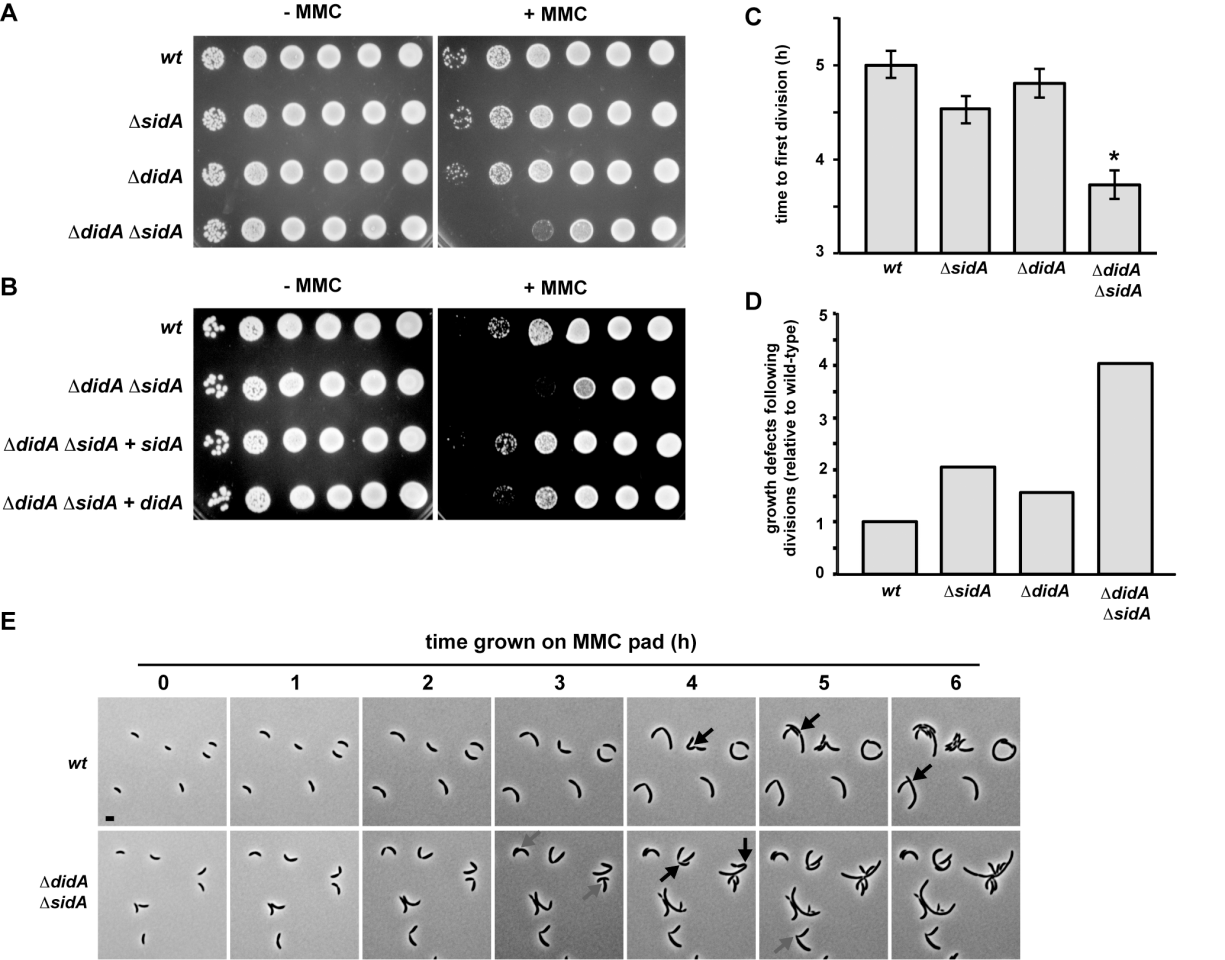
Cells lacking *sidA* and *didA* cannot properly regulate cell division following DNA damage. (A) Wild-type, *ΔsidA*, *ΔdidA*, and *ΔsidAΔdidA* cells were grown to mid-exponential phase and plated in 10-fold dilutions on rich media with or without 0.35 µg/ml MMC. (B) Wild-type and *ΔsidAΔdidA* cells carrying an empty plasmid, and *ΔsidAΔdidA* cells carrying a plasmid with either *sidA* or *didA* driven by its native promoter were plated as in (A). (C–E) Synchronous populations of swarmer cells from the strains in (A) were placed on agarose pads containing rich media and MMC and imaged for 8 hours by time-lapse microscopy. (C) The time to first mid-cell division and (D) the percentage of cells that stopped growing following division relative to the wild type are shown (for criteria on calling divisions and growth cessation, see Text S1). The data in (C) are representative of biological duplicates. The data in (D) are averaged from biological duplicates. Asterisks represent a statistically significant (*p*<0.01) difference relative to the wild type. Error bars represent standard error of the mean (SEM). (E) Representative fields of wild-type and *ΔsidAΔdidA* swarmer cells grown on pads containing MMC at the time points indicated in hours. Black arrows indicate cells that divided. Gray arrows indicate cells arrested for growth following division. Bar, 2 µm.

To test whether DidA is necessary to block cell division following DNA damage, we constructed a strain in which all but the first and last three amino acids of *didA* were deleted. As with a *sidA* deletion strain, *ΔdidA* cells grown on plates containing MMC showed no major viability defect (Figure 3A). However, a strain lacking both *sidA* and *didA* showed a pronounced defect, with a nearly 100-fold decrease in plating efficiency (Figure 3A). This decreased viability was rescued by the presence of either inhibitor on a low-copy plasmid (Figure 3B). These results indicate that SidA and DidA are, to some extent, functionally redundant in blocking cell division following MMC-induced DNA damage.

To better understand the DNA damage sensitivity of *ΔsidAΔdidA* cells, we used time-lapse microscopy to examine synchronous populations of swarmer cells during growth on agarose pads containing MMC. Wild-type swarmer cells did not divide for ∼5 hours on average (Figure 3C), which is significantly longer than the average time to first division of 1.9 hours for wild-type swarmer cells grown on MMC-free pads. On MMC pads, roughly 5% of wild-type cells arrested growth following a cell division event (Figure 3D-3E and Data S2), indicating that division may have been premature or inappropriately executed and was, consequently, lethal. The single deletion strains, *ΔsidA* and *ΔdidA*, also delayed cell division in the presence of MMC; the average time to division was not significantly different than for wild-type cells. These single deletion strains had 1.5–2 times as many growth arrested cells following division events compared to the wild type, although these defects were apparently insufficient to produce a gross viability defect (Figure 3A and 3D). In contrast to the single mutants, *ΔsidAΔdidA* cells lacking both inhibitors divided ∼1.25 hours earlier than wild-type (*p* = 6.9×10^−10^), and four times as many cells exhibited growth defects following a division event (Figure 3C–3E; Data S2). Taken together, our data suggest that the lethality experienced by *ΔsidAΔdidA* cells in the presence of MMC results from an inability to appropriately delay cell division.

### DidA Interacts with the Late-Arriving Divisome Component FtsN

We next sought to investigate how DidA disrupts cell division. We first asked whether DidA interferes with cell division directly, through an interaction with the divisome, or indirectly by inducing the SOS regulon or inhibiting the cell cycle regulator CtrA. To investigate the possibility of indirect mechanisms, we isolated RNA from cells overproducing DidA from a medium-copy plasmid for 45 minutes and compared it on DNA microarrays to RNA from similarly treated cells grown in the absence of inducer. No significant gene expression changes were observed in the SOS or CtrA regulons (Data S1B) suggesting that DidA acts post-transcriptionally, and possibly directly, to inhibit cell division.

**Figure 4:**
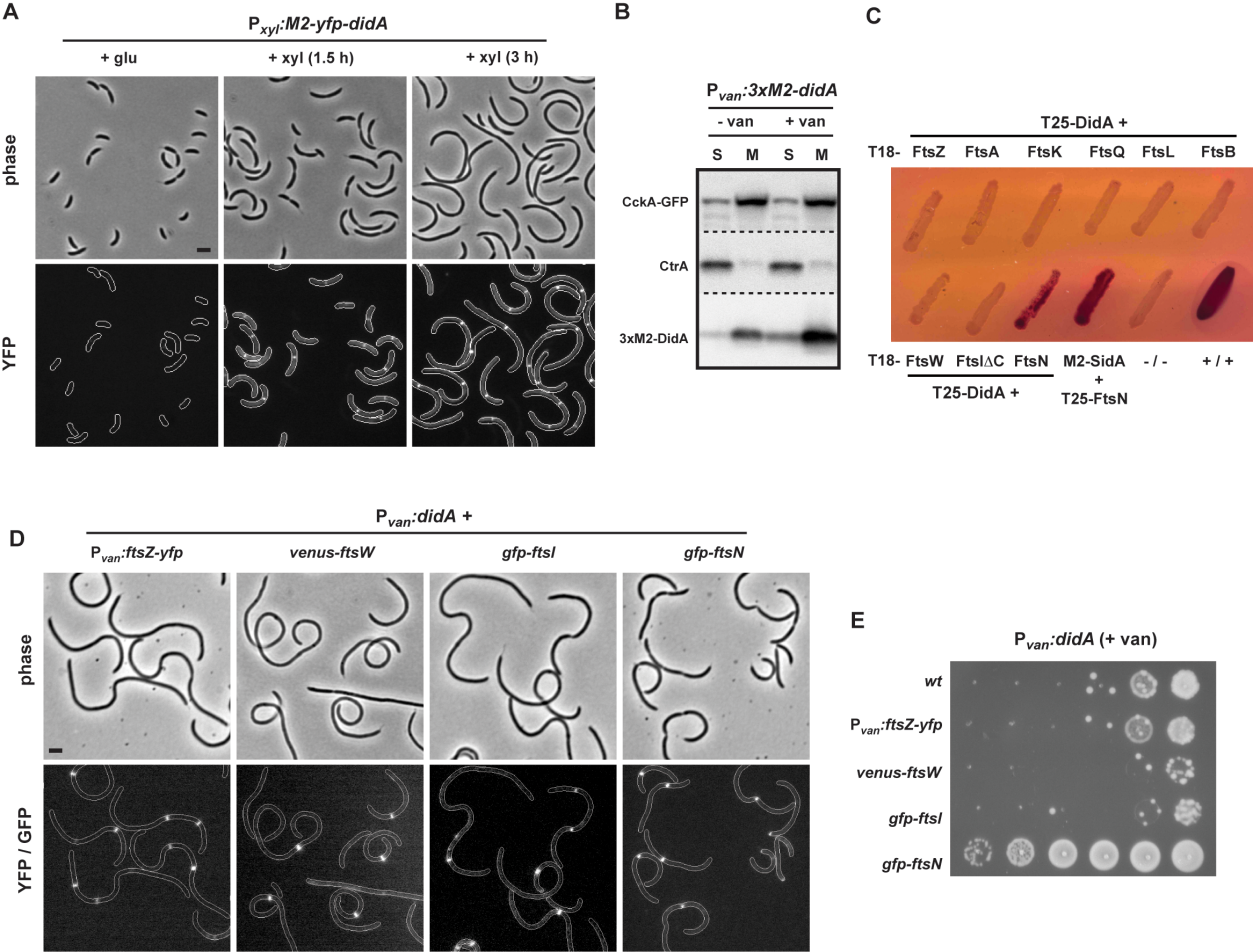
DidA is a small, inner membrane protein that interacts with FtsN. (A) The subcellular localization of DidA was examined in a strain expressing *M2-yfp-didA* from the xylose-inducible promoter P*_xyl_* on a low-copy plasmid. Cells were grown to mid-exponential phase in rich media with glucose and then shifted to xylose. At the times indicated, cells were imaged by phase and epifluorescent microscopy. In the fluorescent micrographs, cell boundaries were added after imaging. (B) Subcellular fractionation of cells overexpressing *3×M2-didA* from the P*_van_* promoter on a medium-copy plasmid for 1.5 hours and expressing the transmembrane protein *cckA-gfp* from P*_cckA_* on the chromosome. Samples were fractionated into soluble (S) and membrane (M) fractions and analyzed by Western blot. The membrane was cut into three pieces, indicated by dashed lines, and probed with antibodies specific for the GFP, CtrA, or M2 epitope. (C) Bacterial two-hybrid analysis of interactions between T25-DidA and cell division proteins fused to T18, as indicated. The FtsIΔC construct lacking the C-terminal catalytic domain previously showed interactions with FtsW and FtsN as expected, unlike the full-length version of FtsI [13]. The interacting pair T18-M2-SidA and T25-FtsN was included for comparison. *E. coli* strains harboring each pair of fusions were plated on LB, and colonies were restruck on MacConkey plates containing maltose. Red streaks indicate positive interactions. −/− indicates empty vectors negative control, +/+ indicates the zip/zip fusions used as a positive control. (D) Subcellular localization of FtsZ, FtsW, FtsI, and FtsN were examined in strains expressing *ftsZ-yfp* from the chromosomal P*_van_* promoter, or *venus-ftsW*, *gfp-ftsI* or *gfp-ftsN* from its native chromosomal locus. Each strain was transformed with a medium-copy plasmid expressing *didA* from the P*_van_* promoter. Strains were grown to mid-exponential phase and samples imaged by phase and epifluorescent microscopy after addition of vanillate for 4.5 hours. In the fluorescent images, cell outlines were drawn based on the phase micrographs. Bar, 2 µm. (E) Strains from (D) were grown to mid-exponential phase and 10-fold serial dilutions were plated on rich media supplemented with vanillate to induce *didA* expression.

To further explore how DidA inhibits cell division, we examined its subcellular localization. In predivisional cells, the major components of the cell division machinery are located at mid-cell [2] where they synthesize a septum and drive invagination of the cell envelope. To assess DidA localization, we transformed wild-type cells with a low-copy plasmid harboring an *M2-yfp-didA* fusion under the control of a xylose-inducible promoter. After induction for 3 hours, cells became filamentous indicating that the YFP-DidA fusion inhibits cell division (Figure 4A). Notably, YFP-DidA foci were frequently observed at pinch sites near mid-cell (Figure 4A) placing it in close proximity to the cell division machinery. Further, fractionation of cells overproducing 3×M2-DidA indicated that DidA is strongly enriched in the membrane where many of the middle- and late-arriving cell division components also reside (Figure 4B). These data are consistent with a model whereby DidA inhibits division through an interaction with a component of the divisome.

To test for interactions of DidA with the known set of critical *Caulobacter* cell division components [2], we performed a bacterial two-hybrid analysis as used previously with SidA [13],[16]. Briefly, proteins were fused to either the T18 or T25 subunit of adenylate cyclase and co-expressed in *E. coli*; a protein-protein interaction reconstitutes adenylate cyclase and drives synthesis of cyclic-AMP, causing colonies to appear red on MacConkey agar plates. When expressed from the low-copy plasmid pKT25, a T25-DidA fusion interacted almost exclusively with the late-arriving cell division protein fusion T18-FtsN (Figures 4C and S4A). Identical results were obtained in the reciprocal orientation, with a T18-DidA fusion on the high-copy plasmid pUT18C and individual division proteins produced from pKT25 (Figure S4B). SidA, whose primary target is likely FtsW, also interacts, to some extent, with FtsN (Figure 4C) [13]. In sum, our data suggest that DidA is an integral membrane protein that localizes to mid-cell where it may disrupt cell division through an interaction with FtsN.

FtsN is among the last cell division proteins to arrive at mid-cell prior to cytokinesis. Although its precise function is unknown, FtsN interacts with multiple division proteins and may help stabilize the assembled divisome [16]–[18]. To ask whether DidA destabilizes or blocks assembly of the divisome, we examined the localization of early- and late-arriving division proteins during DidA overproduction. Cells producing fluorescently tagged FtsZ, FtsW, FtsI, or FtsN were transformed with a plasmid for overexpressing *didA* and then grown in the presence of vanillate to induce DidA synthesis. After 4.5 hours of induction, cells expressing *ftsZ-yfp*, *venus-ftsW*, or *gfp-ftsI* were inhibited for cell division, but 89%, 95%, and 85% of cells, respectively, contained fluorescent foci at or near visible pinch sites (Figure 4D). These results indicate that DidA likely does not disrupt the localization of cell division proteins or drive the disassembly of division protein complexes. Additionally, we noted that many cells displayed multiple foci of the FtsZ, FtsW, or FtsI fluorescent fusions suggesting that DidA also does not prevent the formation of new division assemblies.

Intriguingly, cells expressing *gfp-ftsN* were noticeably shorter (12.6±0.65 µm standard error of the mean [SEM]) and more pinched than those expressing *ftsZ-yfp*, *venus*-ftsW, or *gfp-ftsI* (22.8±0.79, 24.7±1.05, 26.1±0.74 µm, respectively) (Figure 4D). Further, cells expressing *gfp-ftsN* robustly formed colonies despite DidA overproduction, in contrast to cells expressing the other fluorescent fusions (Figure 4E), indicating that *gfp-ftsN* functions as a DidA suppressor, possibly by decreasing its affinity for DidA or by stabilizing FtsN and thereby increasing FtsN levels. In either case, these data further support a model in which DidA interacts with FtsN to block cell division, but without disrupting assembly of an intact divisome.

We next sought to determine whether point mutations in FtsN can also suppress the lethality of overproducing DidA. We first constructed a low-copy plasmid on which *3×M2-didA* was transcribed from the IPTG-inducible promoter P*_lac_*. We then used mutagenic PCR to create a library of *ftsN* mutants containing, on average, one nucleotide substitution per coding sequence; these *ftsN* mutants were cloned into a medium-copy plasmid with expression driven by P*_xyl_*. The *didA* expression vector and *ftsN* plasmid library were co-transformed into an *ftsN* depletion strain in which the only chromosomal copy of *ftsN* is transcribed from the P*_van_* locus [19]. Cells were plated in the presence of IPTG to induce 3×M2-DidA, but without vanillate such that only plasmid-produced, mutant FtsN accumulated. From ∼168,000 cells plated, two candidate *ftsN* suppressors were isolated that suppressed the lethality of overproducing DidA. Plasmid sequencing indicated that one clone contained a single mutation, *ftsN(L202P)*, while the other contained two mutations, *ftsN(P156S)* and *ftsN(F252L)*.

**Figure 5:**
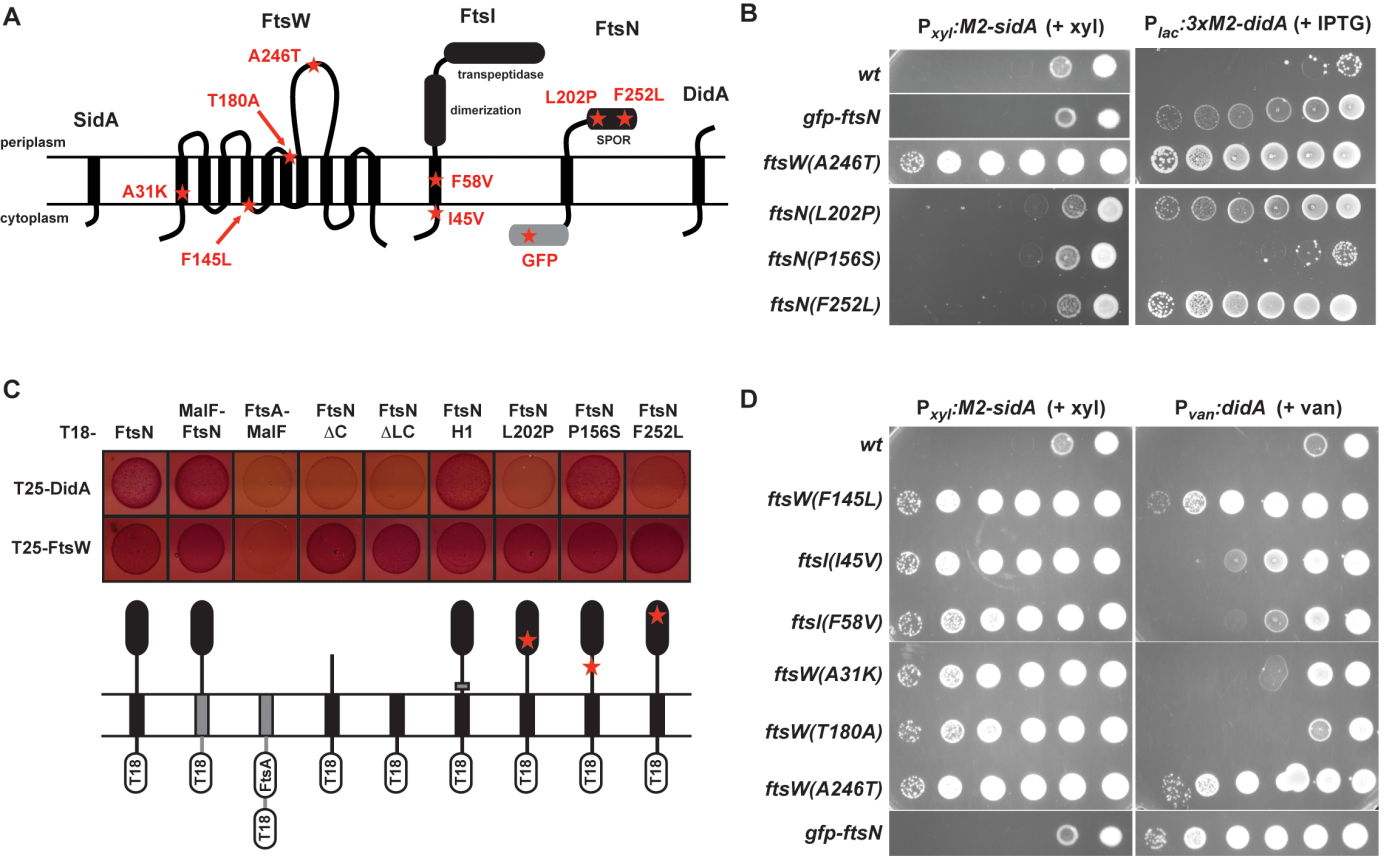
Mutations in the FtsW/FtsI/FtsN complex suppress SidA and DidA overproduction phenotypes. (A) Schematic showing the membrane topology of FtsW, FtsI, FtsN, SidA, and DidA. Missense mutations and the GFP-FtsN fusion that suppress the activities of SidA or DidA, or both, are listed in red. (B) Strains harboring the mutations indicated were transformed with a medium-copy plasmid expressing *M2-sidA* from the P*_xyl_* promoter or a low-copy plasmid expressing *3×M2-didA* from the P*_lac_* promoter. To induce *M2-sidA*, strains were grown in media supplemented with glucose and then plated on media supplemented with xylose. To induce *3×M2-didA*, strains were grown in media without inducer and then plated with IPTG. Each strain was plated in 10-fold dilutions. (C) Bacterial two-hybrid analysis of interactions between T25-DidA or T25-FtsW and T18 fusions to FtsN and the mutants indicated. Below is a graphical representation of each T18 construct. (D) Strains harboring the mutations indicated were transformed with plasmids for inducing M2-SidA or DidA and plated on inducing media.

Each mutation was introduced into an otherwise wild-type chromosome and tested for its ability to suppress 3×M2-DidA overproduction. Only those cells harboring the *ftsN(L202P)* or *ftsN(F252L)* mutation maintained 3×M2-DidA suppression (Figure 5A and 5B), indicating that *ftsN(P156S)* was likely a passenger mutation with *ftsN(F252L)*. Intriguingly, both *bona fide* suppressor mutations reside within the periplasmic, C-terminal “SPOR” domain of FtsN, which may bind peptidoglycan structures within the actively dividing, septal cell wall [19]–[21].

To further explore the regions of FtsN that bind DidA, we tested a series of FtsN truncations and chimeras in the bacterial two-hybrid system (Figure 5C). T25-DidA still interacted with an FtsN construct whose cytoplasmic and transmembrane domains were replaced with the transmembrane domain of the *E. coli* permease MalF, but not with a MalF fusion to the divisome component FtsA. In contrast, the DidA-FtsN interaction was significantly weakened when FtsN constructs lacked either its entire periplasmic portion or the periplasmic SPOR domain alone. We also noted that DidA still interacted robustly with an FtsN construct in which the only known essential domain, located within the periplasmic linker region and denoted “H1” [19], was replaced with an unstructured region of the *Caulobacter* protein SpmX. Collectively, these results suggest that DidA binds the periplasmic SPOR domain of FtsN where the suppressor mutations L202P and F252L reside. Moreover, we found that, when introduced into T18-FtsN, each suppressor mutation strongly reduced the interaction with DidA compared to wild-type FtsN or FtsN(P156S) which, as noted, does not suppress DidA lethality (Figure 5C). Importantly, each of the FtsN mutants tested interacted with FtsW as well as the wild-type FtsN did, indicating that the mutants were properly expressed and folded. In summary, our results suggest that DidA binds the SPOR domain of the late-arriving divisome component FtsN, and the substitutions L202P and F252L in this domain suppress the lethality of overproducing DidA by reducing its affinity for FtsN.

### Mutations in *ftsW* Can Suppress the Division Inhibition Caused by Either SidA or DidA

To further explore the mechanism by which DidA inhibits division, we also screened for spontaneous mutations that suppress the lethality of overproducing DidA. Wild-type cells carrying a medium-copy plasmid expressing *3×M2-didA* from P*_van_* were grown on plates containing vanillate to induce 3×M2-DidA. Because wild-type cells overproducing 3×M2-DidA cannot form colonies (Figure 5B), those rare colonies arising on plates containing vanillate represent strains harboring putative suppressor mutations. From roughly 3×10^7^ plated cells, 34 suppressors were identified, although only one strain retained high levels of functional 3×M2-DidA. Whole genome resequencing identified a putative suppressor mutation in *ftsW*, which would produce the substitution A246T in the predicted large periplasmic loop of FtsW (Figure 5A). This mutation was created *de novo* in a wild-type background and confirmed to suppress the lethality of overproducing DidA (Figure 5B). As noted, no interactions between DidA and FtsW were observed in our two-hybrid analysis. This could be a false negative; alternatively, FtsW(A246T) may suppress DidA overproduction by promoting an activity of FtsW rather than by preventing binding of the inhibitor.

Intriguingly, we had previously found other mutations in *ftsW* that suppress the lethality of overproducing SidA [13]. We therefore reasoned that SidA and DidA may function similarly to inhibit cell division. To explore this possibility, we asked whether the previously identified suppressors of SidA overproduction could also suppress DidA overproduction, and *vice versa* (Figure 5A and 5D). Several mutations primarily suppressed the lethality of only one of the inhibitors. For instance, the FtsW(A31K) strain strongly suppressed overproduction of M2-SidA but not DidA, whereas the strains producing FtsN(L202P) or FtsN(F252L) suppressed the activity of DidA but not M2-SidA. These inhibitor-specific suppressors likely prevent binding of their respective inhibitors (Figures 5C and S5) [13]. The other mutations showed varying abilities to suppress the lethality associated with overproducing either inhibitor. In particular, the strains producing FtsW(F145L) or FtsW(A246T) showed robust suppression of both inhibitors.

The ability of these single substitutions, F145L and A246T, to suppress the lethality of overproducing either SidA or DidA could indicate that the inhibitors share a binding site within FtsW that is disrupted by the suppressor mutations. However, this is unlikely given that (1) DidA binds FtsN, but not FtsW, in our bacterial two-hybrid system, (2) DidA-YFP still localizes to the septum in cells producing FtsW(A246T) (Figure S5A), and (3) M2-SidA binds to FtsW(A246T) to the same extent as it does to wild-type FtsW (Figure S5B). Instead, we hypothesized that the subcomplex of late-arriving division components FtsW, FtsI, and FtsN could exist in one of two states: an active state that promotes constriction of the septum and cell division, and an inactive state that is promoted or stabilized by SidA and DidA. In this model, the suppressor mutations in *ftsW* and *ftsI* promote the active state and thus enable cell division even in the presence of SidA and DidA.

### SidA and DidA Suppressor Mutations Drive Hyperactive Cell Division

**Figure 6:**
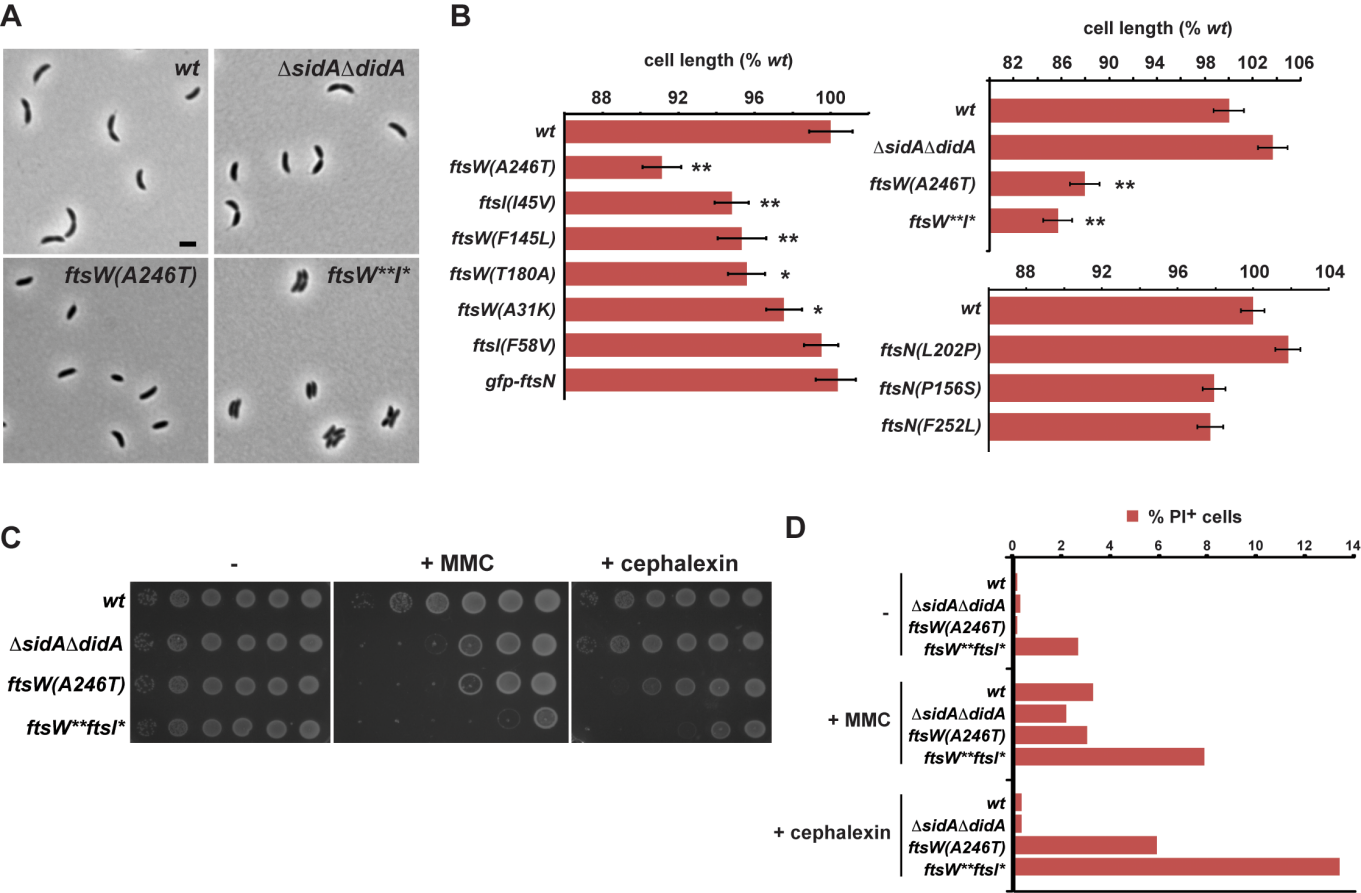
Mutations that suppress *sidA* and *didA* overexpression likely hyperactivate cell division. (A) The strains indicated were grown to mid-exponential phase in rich media and imaged by phase microscopy. Bar, 2 µm. (B) Each strain indicated was grown to mid-exponential phase and average cell length, relative to wild-type, was calculated (all *n*>440). Error bars represent standard error of the mean (SEM), and asterisks indicate *p*<0.01 (*) or *p*<0.0001 (**). The strain denoted ftsW**I* combines the mutations *ftsW(F145L, A246T)* and *ftsI(I45V)*. Separate graphs are shown for cell length measurements made on different days. For raw data, see Data S3. (C) Wild-type, *ΔsidAΔdidA*, *ftsW(A246T)*, and ftsW**I* cells were grown to mid-exponential phase and plated in 10-fold dilutions on rich media containing no additives, 0.35 µg/ml MMC or 6 µg/ml cephalexin. (D) The strains from (C) were grown to mid-exponential phase in rich media and treated with MMC or cephalexin at the concentrations in (C) for 6 hours. PI at 5 µM was added 1.5 hours before imaging. Cells were imaged by phase and fluorescence microscopy; cell lengths and percentage of PI+ cells are shown by bar graphs. For raw data, see Data S3.

If the FtsW(F145L) and FtsW(A246T) mutations promote an active state of a subcomplex of cell division proteins, then cells harboring these mutations, but not producing SidA or DidA, may attempt division earlier than wild-type cells, even in the absence of DNA damage. To explore this possibility, we grew strains harboring one of the suppressor mutations in *ftsW*, *ftsI*, or *ftsN* into mid-exponential phase in rich medium and measured cell lengths in a large population of cells. Indeed, several of the suppressor mutations resulted in cells that were significantly shorter on average than wild-type cells even though their growth rates were not substantially different (Figures 6A, 6B, and S6A–S6C). For *ftsW(A246T)*, we verified that all cell types were shorter, indicating that the mutant strains are not trivially enriched for swarmer cells (Figure S6B). The degree of shortening roughly correlated with the ability to suppress both SidA and DidA activity, as cells harboring the mutations *ftsW(A246T)*, *ftsW(F145L)*, and *ftsI(I45V)* that were best able to suppress both SidA and DidA were also the shortest. Conversely, mutations that only suppressed the activity of one inhibitor were typically not shorter than wild-type. We found that Δ*sidA*Δ*didA* cells were also not shorter than wild-type cells. Taken together, these results are consistent with a model in which suppressors exhibiting short cell phenotypes harbor gain-of-activity mutations rather than simply being defective for SidA or DidA binding.

Given that the *ftsW(A246T)* mutation renders cells insensitive to SidA and DidA, this suppressor strain should also divide earlier than wild-type cells in the presence of MMC like the *ΔsidAΔdidA* deletion strain. To test this prediction, we grew populations of wild-type and *ftsW(A246T)* cells on agarose pads containing MMC and measured the time to first division by time-lapse microscopy. The *ftsW(A246T)* cells divided an average of 35 minutes earlier than wild-type cells and showed a 5-fold increase in the fraction of cells that stopped growing following a division event (Figure S7A and S7B). Accordingly, *ftsW(A246T)* cells showed a similar sensitivity on MMC plates as observed with the *ΔsidAΔdidA* strain (Figure 6C).

Although the *ftsW(A246T)* and *ΔsidAΔdidA* strains behave similarly in the presence of MMC, only the *ftsW(A246T)* strain exhibited a short cell phenotype when grown without MMC (Figure 6A and 6B). The *ftsW(A246T)* cells grew at approximately the same rate as wild-type cells in the absence of MMC; these cells are born shorter than wild-type cells, but also divide when shorter than wild-type cells resulting in nearly identical division cycle times (Figure S6B–6D). The short cell phenotype of this strain in the absence of MMC suggested that FtsW(A246T) harbors increased cell division activity, and has a propensity to divide early, compared to wild-type and *ΔsidAΔdidA* cells. To further explore this activity, we combined the three suppressor mutations conferring the shortest cell length phenotypes, *ftsW(A246T)*, *ftsI(I45V)*, and *ftsW(F145L)*, engineering each on the chromosome of a single strain. When grown in the absence of MMC, this triple mutant, denoted ftsW**I*, was slightly shorter than the single *ftsW(A246T)* mutant and exhibited an increased sensitivity to MMC compared to the *ftsW(A246T)* and *ΔsidAΔdidA* strains (Figure 6C). These results suggest that the triple mutant likely harbors increased activity relative to the single *ftsW(A246T)* mutant that alone causes cells to attempt divisions more hyperactively both in the presence and absence of MMC.

We also noticed that the ftsW**I* strain grew more slowly than wild-type or *ftsW(A246T)* cells in liquid cultures (Figure S6C). Because FtsW and FtsI participate in septal cell wall synthesis, we suspected that this growth phenotype may result from premature or misregulated cell division events that compromise cell wall integrity. To test this possibility, we stained wild-type, *ΔsidAΔdidA*, *ftsW(A246T)*, and ftsW**I* cells with propidium iodide (PI), a dye that binds nucleic acids, but only if the cell envelope is compromised (Figure 6D). Whereas wild-type, *ΔsidAΔdidA*, and *ftsW(A246T)* cells were rarely (0.1%–0.3% of cells) stained by PI, 2.6% of ftsW**I* cells were PI-positive. Given these results, we also tested whether the *ftsW(A246T)* and ftsW**I* strains were more sensitive than wild type when treated with cephalexin, which interferes with septal cell wall synthesis by blocking the transpeptidase activity of FtsI. Cephalexin does not directly cause DNA damage, and cells treated with cephalexin showed no noticeable induction of *sidA* or *didA* (Figure S8). It was thus not surprising that *ΔsidAΔdidA* cells showed no growth defect compared to wild-type when grown on plates containing a low dose of cephalexin that does not significantly perturb growth or division in wild-type cells (Figure 6C and 6D). In contrast, the *ftsW(A246T)* and ftsW**I* strains each exhibited cephalexin sensitivity, particularly ftsW**I* (Figure 6C). When grown as liquid cultures with cephalexin, the *ftsW(A246T)* and ftsW**I* strains had 18- and 48-fold, respectively, more PI-positive cells than wild-type (Figures 6D and S9). By contrast, there was not a similar enrichment of PI-positive cells in the *ftsW(A246T)* and ftsW**I* strains following an MMC treatment. Furthermore, while the average lengths of cells from the suppressor strains were decreased relative to wild type in MMC, likely due to premature divisions, they were longer in cephalexin, indicating a decreased ability to divide ().

In sum, cells harboring the mutation *ftsW(A246T)*, either alone or in combination with *ftsI(I45V)* and *ftsW(F145L)*, exhibit cell wall defects and are more sensitive to a cell wall synthesis inhibitor. Importantly, cells lacking *sidA* and *didA* do not exhibit these same cell wall defects. These results are consistent with a model in which the mutations identified in *ftsW* and *ftsI* do not suppress SidA and DidA by simply preventing the binding of these inhibitors, but instead affect septal cell wall synthesis and increase the propensity of cells to initiate cell division.

### *sidA* and *didA* Are Differentially Regulated

Our identification of *didA* indicates that *Caulobacter* cells have an SOS-independent mechanism for sensing and responding to DNA damage. To explore this alternative, damage-inducible pathway, we first asked whether *didA* is induced specifically by DNA damage or more generally by cellular stress. Cells harboring a *didA-3×M2* fusion at the native *didA* locus were treated with a variety of stresses, but the only conditions leading to a significant induction of *didA* were DNA damaging agents (Figure S10).

**Figure 7:**
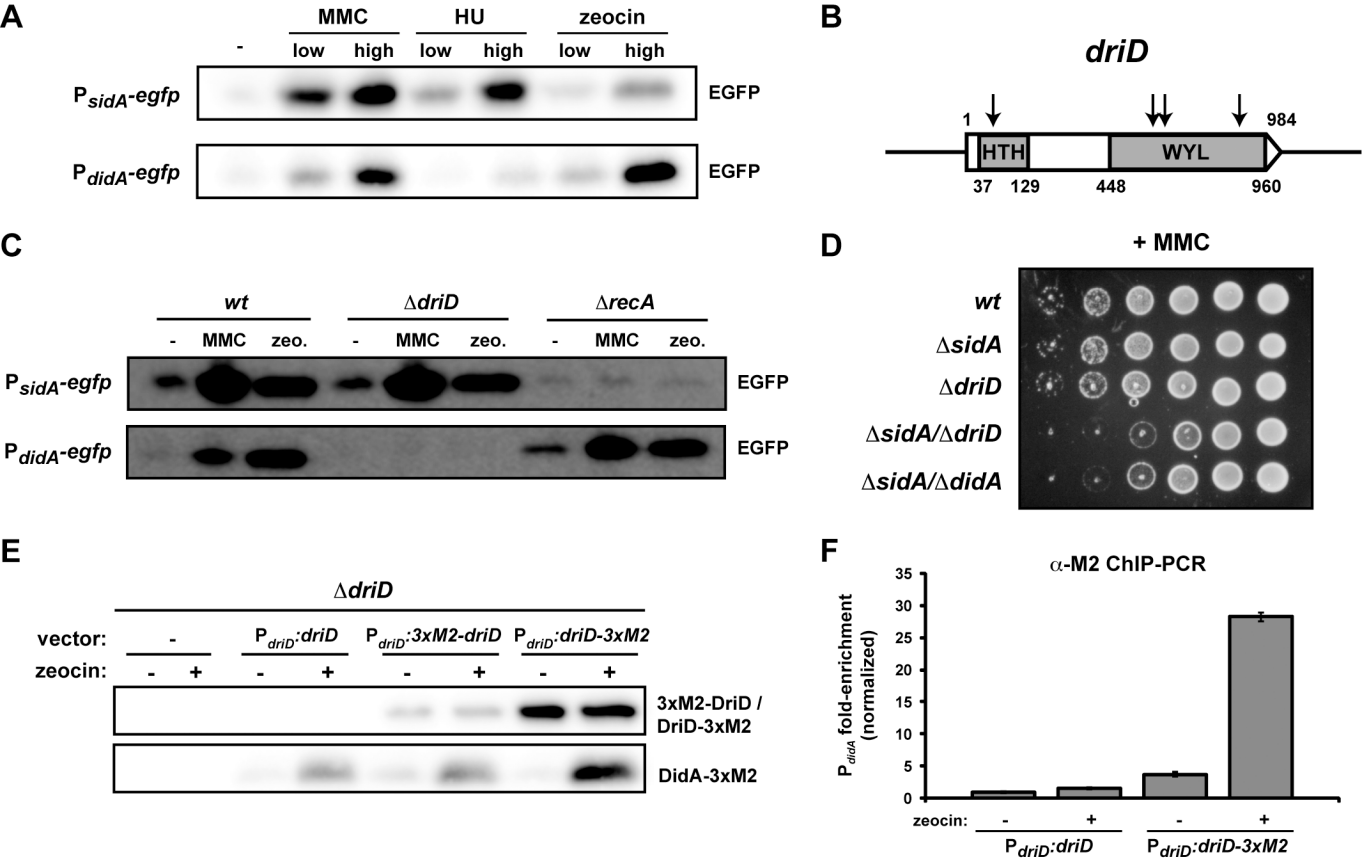
DriD directly activates *didA*. (A) Wild-type cells harboring low-copy plasmids expressing *egfp* from the *sidA* or *didA* promoters were treated with MMC (0.5 and 3 µg/ml), hydroxyurea (HU; 0.5 and 3 mg/ml) or zeocin (2.5 and 15 µg/ml) and then analyzed by Western blot using an α-GFP antibody. (B) Diagram of *driD* indicating the predicted helix-turn-helix (HTH) and WYL domains. Arrows indicate transposon insertion sites in the genetic screen that identified *driD*. (C) Wild-type, *ΔdriD*, and *ΔrecA* cells were transformed with the P*_sidA_* and P*_didA_* reporter plasmids from (A) and treated with 3 µg/ml MMC or 15 µg/ml zeocin for 1 hour. Samples were analyzed by Western blot using an α-GFP antibody. (D) 10-fold serial dilutions of the strains indicated were grown on plates containing 0.35 µg/ml MMC. (E) *ΔdriD* cells carrying a low-copy plasmid producing a control construct (P*_xyl_-ftsW-egfp*), untagged DidA, or DidA fused at either its N- or C-terminal end to a 3×M2 tag and expressed from the *didA* promoter were treated with 15 µg/ml zeocin for 45 minutes. Samples were analyzed by Western blot using an α-FLAG/M2 antibody. (F) *ΔdriD* cells carrying a low-copy plasmid expressing either *driD* or *driD-3×M2* from the *driD* promoter were treated with 15 µg/ml zeocin for 45 minutes. DriD was immunoprecipitated with an α-FLAG/M2 antibody and promoter occupancy was analyzed by quantitative PCR using primers specific for P*_didA_*. Fold-enrichment values were normalized relative to the enrichment of a region within the coding sequence of *ruvA*. For raw data, see Data S3.

To further examine *didA* induction and compare it to *sidA* induction, we transformed wild-type cells with plasmids harboring a transcriptional fusion of *egfp* to either the *sidA* or *didA* promoter and then treated each strain with (i) MMC, an alkylating agent that forms single-stranded DNA adducts and double-stranded cross-links, (ii) hydroxyurea, which depletes the dNTP pool by inhibiting ribonucleotide reductase and stalls replication forks, thereby mimicking a consequence of DNA damage, or (iii) zeocin, which directly cleaves DNA, creating double-strand breaks. Western blots for GFP indicated that MMC strongly induced both *sidA* and *didA* (Figure 7A). In contrast, hydroxyurea drove induction of P*_sidA_*, but not P*_didA_*, even at high doses. Conversely, zeocin strongly induced P*_didA_*, but only weakly induced P*_sidA_*. These data indicate that the SOS-independent induction of *didA* involves a signal or DNA structure that is distinct from the ssDNA-RecA-dependent induction of *sidA*. In particular, the strong induction of P*_didA_* by zeocin suggests that the signal may be a DNA structure associated with the presence or repair of double strand breaks, which also arise following MMC exposure [22].

### Identification of *driD*, an SOS-Independent, DNA Damage-Induced Transcription Factor

We devised a genetic screen to identify factors involved in *didA* induction. In a Δ*didA* background, we fused the *didA* promoter to *lacZ* and integrated this reporter construct at the *hfaB* locus, a region of low transcription. When grown in the presence of X-gal, colonies with high P*_didA_* activity should express *lacZ* and appear blue while those with low P*_didA_* activity should appear white. We mutagenized this strain using a Tn5 transposon and screened for mutants on X-gal plates containing MMC. We chose a dose of MMC low enough to allow colony formation, but high enough to induce *didA* induction resulting in blue colonies. We screened ∼26,000 colonies and isolated nine white colonies; five of these colonies had Tn5 insertions in the P*_didA_*-*lacZ* reporter while the remaining four contained insertions in the coding region of CCNA_01151 (Figure 7B). This gene is annotated as a DeoR-family transcriptional regulator and is predicted to encode an N-terminal DNA-binding domain with a C-terminal ligand-binding domain (“WYL domain,” Pfam domain 13280). Each of the four insertions in CCNA_01151 was unique with one occurring in the DNA-binding domain and the other three in the C-terminal WYL domain. We named CCNA_01151 *driD* (for DeoR inducer of *didA*).

To confirm that DriD induces *didA*, we constructed a strain in which all of *driD* except the first three and last ten amino acids were deleted. We then transformed wild-type, Δ*driD*, and Δ*recA* cells with low-copy plasmids harboring P*_sidA_*-*egfp* or P*_didA_*-*egfp* reporters and monitored the inducibility of each promoter following MMC or zeocin treatment by Western blotting with α-GFP (Figure 7C). As expected, *sidA* induction by either DNA damaging agent requires the SOS regulator gene *recA* but is unaffected in cells lacking *driD*. In contrast, *didA* induction occurs in Δ*recA* cells but not in cells lacking *driD*. These results confirm the SOS-independent inducibility of *didA* and indicate that *driD* is required for *didA* induction. We also tested whether the *driD* deletion behaves like a *didA* deletion with respect to MMC sensitivity (Figure 7D). Indeed, cells lacking both *sidA* and *driD* exhibited a roughly 100-fold reduction in viability when grown on MMC plates, compared to the wild type and strains lacking either *sidA* or *driD*. A nearly identical defect was observed when combining *sidA* and *didA* deletions, further supporting a model whereby DriD drives *didA* induction.

We next sought to complement our *driD* deletion by introducing low-copy plasmids containing P*_driD_* fused to wild-type *driD* or a copy of *driD* encoding an N- or C-terminal fusion to the 3×M2 epitope; each strain also harbored a chromosomal *didA-3×M2* reporter to assess DriD activity. Whereas cells carrying an empty vector were unable to induce *didA* when treated with zeocin, cells with wild-type or either tagged version of *driD* were able to induce *didA* (Figure 7E, bottom panel). Additionally, we noted that the levels of both 3×M2-tagged DriD constructs remained unchanged following zeocin treatment (Figure 7E, top panel) indicating that DriD activity is regulated post-translationally.

Finally, to determine whether DriD directly activates *didA*, we assessed DriD occupancy at P*_didA_* using chromatin immunoprecipitation (ChIP) followed by quantitative PCR. Cells expressing *driD* or *driD-3×F* from a plasmid as the only copy of *driD* were treated with zeocin for 45 minutes or left untreated and then subjected to ChIP using an α-FLAG/M2 antibody (Figure 7F). P*_didA_* was minimally enriched (normalized IP output/input) in the immunoprecipitate of cells expressing untagged DriD. In cells expressing *driD-3×M2*, P*_didA_* was enriched roughly 3.5-fold in the absence of zeocin and nearly 30-fold following zeocin treatment. Taken together, our data suggest that DriD is a direct, positive regulator of *didA* induction that is enriched at the *didA* promoter following certain types of DNA damage, including double-strand breaks.

## Discussion

### SOS-Independent Regulation of the DNA Damage Response

During episodes of DNA damage, cells often use checkpoint systems to transiently inhibit the cell cycle and prevent cell division [23]. In bacteria, the regulatory paradigm for responding to DNA damage has long been the *E. coli* SOS system in which cleavage of the repressor LexA drives the transcription of DNA repair genes and the cell division inhibitor *sulA* [8],[9],[24]. SOS-induced division inhibitors have subsequently been identified in a range of other bacteria, including *sulA* homologs in γ-proteobacteria and the unrelated genes *yneA*, *divS*, *chiZ*, and *sidA* in various other species [10]–[13],[25]. Although these SOS-dependent regulators are often assumed to be the primary, or even sole, mechanism for inhibiting division post-damage, there have been hints of SOS-independent division regulation. For instance, in *E. coli, Bacillus subtilis*, and *Caulobacter*, cells lacking their SOS-induced inhibitors or unable to induce an SOS response can still become filamentous following DNA damage indicating an alternative means of blocking cell division [26]–[30]. However, to the best of our knowledge, no damage-induced, SOS-independent division regulators have been previously documented. Here, we identified *didA* in *Caulobacter* as one such regulator.

How do *Caulobacter* cells recognize and respond to DNA damage to induce *didA* if not through the canonical derepression of SOS genes? DriD is a direct transcriptional activator of *didA*, but how does DriD sense DNA damage? One possibility is that DriD somehow senses the accumulation of the SOS signal ssDNA, which stimulates RecA to trigger the autocatalytic cleavage of LexA [31]–[33]. Another protein, such as the RecA homolog RadA, could also recognize ssDNA, but ultimately activate DriD. However, this scenario is unlikely given the differential induction of *sidA* and *didA* following exposure to DNA damaging agents with distinct mechanisms. Alternatively, a DNA damage sensor unrelated to RecA could recognize a distinct type of DNA damage or DNA structure. For instance, the strong induction of *didA* following zeocin exposure could indicate that the *didA* induction machinery recognizes double-strand breaks. In *B. subtilis*, the diadenylate cyclase DisA monitors genome integrity and may recognize branched DNA structures that arise during the recombination-based repair of double-strand breaks [34]. When paused at such DNA structures, DisA is prevented from synthesizing cyclic-di-AMP (c-di-AMP), a diffusible molecule required for the activation of the transcription factor Spo0A, thereby coupling DNA damage with transcription [34]–[36]. It remains unclear precisely how c-di-AMP affects Spo0A activity in *B. subtilis* and whether a c-di-AMP-based response to DNA damage extends to other organisms. Nonetheless, *didA* transcription could follow a similar regulatory strategy that relies on c-di-AMP, or another damage-regulated second messenger. This is a particularly attractive hypothesis since DriD, annotated as a DeoR-family transcription factor has a C-terminal domain predicted to bind a small molecule. Additionally, we found that DriD levels did not change following zeocin treatment, but occupancy and activation of the P*_didA_* promoter by DriD increased significantly. This finding suggests that DriD activity is post-translationally regulated in a DNA damage-dependent manner, so identification of the putative DriD ligand will be a critical next step.

### The Execution and Regulation of Cell Division

Many cell division inhibitors, including *E. coli* SulA, block cell division by disrupting FtsZ polymerization. FtsZ is an effective target as it recruits most other cell division proteins. However, neither DidA nor SidA affect the assembly of FtsZ rings in *Caulobacter* or stimulate Z-ring disassembly, and neither inhibitor prevents the assembly of downstream divisome components. Instead, these inhibitors appear to block cell division by targeting FtsW, FtsI, and FtsN within the assembled divisome. Bacterial two-hybrid studies indicated that DidA interacts with FtsN. Additionally, several point mutations in *ftsN* diminish the interaction with DidA and suppress the effects of overproducing DidA, supporting a model in which DidA inhibits cell division by binding directly to FtsN, although it remains formally possible that an *E. coli* divisome protein bridges DidA and FtsN in the two-hybrid analysis. SidA interacts with FtsW and FtsN in the bacterial two-hybrid system, and the lethality of overproducing SidA can be suppressed by mutations in either FtsW or FtsI [13]. Although DidA and SidA bind different proteins, these two inhibitors likely inhibit division in similar ways as two mutations in *ftsW*, and one in *ftsI*, can suppress the effects of overproducing either SidA or DidA.

FtsW, FtsI, and FtsN are among the last essential proteins recruited to the cytokinetic ring. These proteins physically interact with each other and likely form a subcomplex within the divisome that drives the synthesis and remodeling of the septal cell wall [2],[37]–[39]. Although its precise biochemical function is unknown, FtsW somehow contributes to septal cell wall synthesis, as does FtsI, which harbors peptidoglycan transpeptidase activity [40],[41]. The function of FtsN is also unclear, although in *Caulobacter* its essential activity is located within a periplasmic linker domain [19]. In both *Caulobacter* and *E. coli*, FtsN recruits proteins involved in cell wall remodeling to the division site [42]–[46], and *E. coli* FtsN has been suggested to stimulate the transpeptidase activity of PBP1B and could act similarly on FtsI [47].

How do single mutations in FtsW and FtsI prevent the inhibition of cell division by both SidA and DidA? One possibility is that these mutations reduce the affinities of SidA and DidA for their division protein targets. However, SidA binding to FtsW was unaffected by the A246T mutation and DidA binds FtsN, not FtsW or FtsI, in our bacterial two-hybrid system. Another possibility is that SidA and DidA block the recruitment of even later arriving proteins. As noted, FtsN may help recruit cell wall remodeling factors such as the peptidase DipM and the peptidoglycan amidase AmiC [43],[45]. Although the genes encoding such proteins are individually dispensable, it is formally possible that SidA and DidA disrupt the recruitment of multiple peptidoglycan remodeling factors, thereby preventing division. However, given that the inhibitory activity of both SidA and DidA can be suppressed by mutations in FtsW and FtsI, this model seems unlikely.

**Figure 8:**
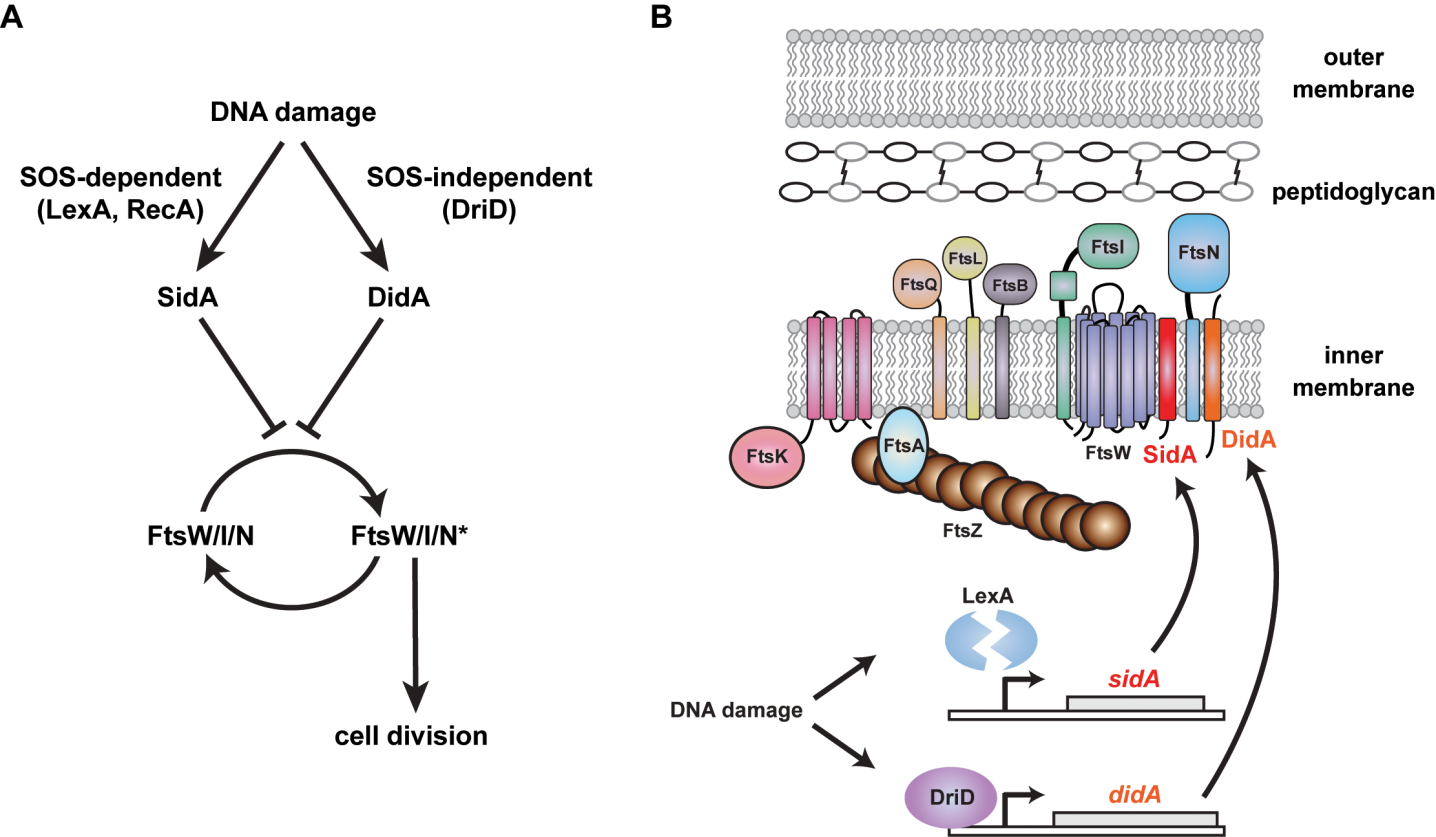
Two independent pathways regulate cell division in *Caulobacter* following DNA damage. (A–B) Two cell division inhibitors are induced following DNA damage in *Caulobacter*. *sidA* is induced by cleavage of the SOS repressor LexA while *didA* is induced by DriD. SidA and DidA are small transmembrane proteins that can block cell division by preventing the divisome subcomplex FtsW/I/N from assuming an active state, designated FtsW/I/N*. FtsW/I/N* could promote division by enhancing peptidoglycan synthesis and remodeling, by triggering FtsZ constriction, or by coordinating these activities.

Instead, we favor a model in which the FtsW/FtsI/FtsN subcomplex exists in two states: an inactive state that is promoted by SidA or DidA, and an active state that drives septal peptidoglycan synthesis and cytokinesis (Figure 8A). We propose that the mutations that suppress both SidA and DidA, such as FtsW(A246T), may lock FtsW/FtsI/FtsN in the active state allowing cells to bypass the block in division normally caused by an accumulation of these inhibitors. On their own, these suppressor mutations cause cells to initiate division hyperactively. In support of this model, cells with the suppressing mutations were reproducibly shorter than wild-type cells (Figure 6A and 6B), likely because they divide at a slightly earlier stage of the cell cycle. Additionally, cells producing FtsW(A246T) or both FtsW(F145L, A246T) and FtsI(I45V) were sensitive to cephalexin, a cell wall synthesis inhibitor, and exhibited compromised cell envelope integrity. Importantly, Δ*sidA*Δ*didA* cells did not exhibit increased sensitivity to cephalexin, further supporting the notion that these mutations in FtsW and FtsI do not simply prevent SidA and DidA binding, but rather increase a cell wall synthesis activity.

Taken together, our results suggest that the DNA damage-induced division inhibitors in *Caulobacter* target the FtsW/FtsI/FtsN subcomplex to block constriction of the division machinery and cell envelope. Precisely how SidA and DidA block division is not yet clear, in part because the execution of cytokinesis remains poorly characterized at a molecular level. The synthesis of septal cell wall material could provide the force and directionality for cellular constriction, with FtsZ required mainly for mid-cell positioning of division proteins. This model is supported by recent data showing that FtsZ often dissociates from the divisome before compartmentalization occurs, indicating that cell wall synthesis may provide the constrictive force for cell division [48]. In such a case, SidA and DidA could prevent division by blocking a critical or rate-limiting peptidoglycan modifying activity of the FtsW/FtsI/FtsN subcomplex. As noted, the suppressor mutants in *ftsW* such as A246T that bypass both SidA and DidA are, on their own, prone to disruption of cell envelope integrity. Their sensitivity to cephalexin could result from certain cell wall synthesis or remodeling activities continuing without concurrent activation of the FtsI transpeptidase domain. As an alternative to this cell wall-centric model for cytokinesis, GTP hydrolysis by the FtsZ ring may provide the energy for, and directionality of, constriction, effectively pulling the rest of the cytokinetic ring along with it [49]. Assembly or activity of the FtsW/FtsI/FtsN subcomplex could somehow trigger FtsZ constriction, and the inhibitors SidA and DidA may block this step of division. Finally, it is possible that Z-ring constriction and septum synthesis combine to drive cytokinesis. As FtsW, FtsI, and FtsN are transmembrane proteins with cytoplasmic and periplasmic domains, they could coordinate the Z-ring and nascent septum, with SidA and DidA disrupting this coordination. Distinguishing between these various models for cytokinesis and elucidating the precise mechanisms of action for SidA and DidA will ultimately require more detailed studies of the FtsW/I/N subcomplex; the mutants identified here, such as FtsW(A246T), may prove particularly useful in these efforts.

### Final Perspectives

Our results (i) reveal an SOS-independent mechanism for inhibiting cell division in *Caulobacter* and (ii) highlight the FtsW/FtsI/FtsN subcomplex as an important regulatory node in the control of cell division. Following certain types of DNA damage, DidA and SidA appear to function together to prevent inappropriate cell divisions (Figure 8). Such redundancy may afford cells with a fail-safe survival mechanism. In addition, SidA and DidA are differentially induced following different types of DNA damage, providing independent routes to the inhibition of cell division under different conditions. Also, we note that although cells lacking both *sidA* and *didA* divide prematurely during DNA damage, many still filament to some degree, suggesting that yet other mechanisms of division inhibition exist in *Caulobacter*. Finally, we note that DidA is the latest in a growing class of small, stress-induced membrane proteins that play critical regulatory roles [50],[51]. These proteins are often missed or incorrectly annotated in genome sequences, but many, like SidA and DidA, clearly play critical roles in regulating cellular processes, including cell division.

## Materials and Methods

### Strains, Plasmids, and Growth Conditions

Strains and plasmids used in this study are listed in Table S1 with construction details and growth conditions provided in Text S1.

### Synchronization

Synchronous *Caulobacter* populations were obtained by centrifugation over a Percoll density gradient as previously described [52]. Following synchronization of the *ftsW(A246T)* strain, we noticed that 21% of cells (two biological replicates) were unable to form microcolonies on plain PYE agarose pads compared to 2% for wild-type cells. Because of this sensitivity to the synchronization procedure, *ftsW(A246T)* cells and other suppressors were imaged by time-lapse microscopy following growth in mixed cultures.

### DNA Microarrays

RNA expression profiling was done as described [53]. Expression experiments were performed in duplicate and the results for each gene were averaged.

### Immunoblots and Biochemical Fractionations

Samples for immunoblots were normalized in sample buffer to 0.5 OD_600_/50 µl, resolved on 12% sodium dodecyl sulfate-polyacrylamide gels and transferred to polyvinylidene difluoride transfer membrane (Pierce). Membranes were probed with polyclonal rabbit α-CtrA, α–DivL, α–LacZ (Rockland Scientific), and α-GFP (Invitrogen) at a 1∶5,000 dilution and monoclonal mouse α-FLAG (Sigma) at a 1∶3,000 dilution. Secondary HRP-conjugated α-rabbit (Pierce) or α-mouse (Pierce) were used at a 1∶5,000 dilution. Blots were visualized by chemiluminescence; raw black-and-white images were inverted for display. Biochemical fractionation was performed as described [13].

### Microscopy

All phase contrast images were acquired on a Zeiss Observer Z1 microscope with a 100×/1.4 oil immersion objective and an LED-based Colibri illumination system. For additional information on image analysis and time-lapse microscopy, see Text S1.

### Bacterial Two-Hybrid Analysis

Two-hybrid complementation assays were performed essentially as described [16]. BTH101 cells harboring plasmids with the T25 and T18 fusion constructs were grown to single colonies on LB agar plates and restruck or spotted on MacConkey agar plates supplemented with maltose for imaging.

### *ftsN* Mutagenesis Screen

The *ftsN* mutagenesis PCR reaction contained 21 µl 3M Betaine, 1 µl DMSO, 5 µl 10× Taq buffer (Invitrogen), 1.5 µl 50 mM MgCl_2_, 4 µl dNTPs, 0.2 µl primers, 50 ng genomic DNA, 2 µl mutagenesis buffer (100 mM dCTP, 100 mM dTTP, 50 mM MgCl_2_, 500 mM MnCl_2_), 0.3 µl Taq polymerase (Invitrogen), and water to 50 µl. The PCR reaction was incubated at 95°C for 5 minutes followed by 35 cycles of 95°C for 1 minute, 58°C for 1 minute, and 72°C for 3 minutes with a final extension of 72°C for 10 minutes. The mutant *ftsN* library was then cloned into a medium-copy plasmid downstream of the xylose-inducible promoter.

An *ftsN* depletion strain harboring a low-copy plasmid expressing *3×M2-didA* from P*_lac_* was transformed with a medium-copy plasmid expressing the mutant *ftsN* library from P*_xyl_* and grown on plates containing oxytetracycline, kanamycin, and 75 or 100 µM IPTG. The medium-copy kanamycin-resistant plasmids from suppressor colonies were isolated and retested in a clean *ftsN* depletion background for their ability to suppress *3×M2-didA* overexpression from the IPTG-inducible low-copy plasmid. *ftsN* mutations in suppressor plasmids were identified by Sanger sequencing.

### Identification of DidA Overproduction Suppressors

Wild-type cells were transformed with a P*_van_:3×M2-didA* overproduction plasmid and plated on PYE agar in absence of vanillate to allow colony formation. Single colonies were grown overnight in PYE and plated on PYE agar supplemented with vanillate at roughly 2×10^6^ colony forming units per 10 cm plate. Rare colonies were grown overnight in PYE supplemented with vanillate and samples were taken for immunoblots, plasmid preparations, and archiving. To isolate chromosomal suppressor mutations and eliminate mutations arising in the *3×M2-didA* overproduction plasmid, we screened for colonies that met two criteria. (1) We used immunoblotting to check that 3×M2-DidA production in each suppressor strain was similar to that seen in wild-type cells transformed with the same plasmid and grown in vanillate for 1.5 h. (2) Plasmids from the suppressor strains were transformed into wild-type cells and plated on PYE agar supplemented with or without vanillate. The presence of thousands of colonies on plain plates and few colonies on vanillate indicated a functional plasmid. The mutation in the *ftsW(A246T)* suppressor strain was identified by whole genome resequencing.

### Screen for Activators of *didA* Expression

Cells expressing *lacZ* from P*_didA_* at the *hfaB* locus in a Δ*didA* background were mutagenized with the EZ-Tn5 transposome (Epicentre) and grown on plates containing kanamycin and 20 µg/ml X-gal. Colonies appearing white were isolated and tested for low or undetectable levels of full-length LacZ by western blot with α-LacZ antibodies. Transposon insertion mutations were identified as described (Epicentre, TSM08KR protocol) by rescue cloning with *pir-116* electrocompetent *E. coli* cells (Epicentre).

### ChIP and Quantitative PCR Analysis

ChIP was performed as detailed in Text S1. Quantitative PCR was performed with the dye SYBR Green (Roche) on a Lightcycler 480 system (Roche). Each reaction contained 5 µl SYBR Green Master, 1 µl DNA (diluted 1∶500 for pre-ChIP input DNA, and 1∶20 for post-ChIP output DNA), 0.5 µl primer mix at 10 µM, and 3.5 µl nuclease-free water. Primers amplifying a product within the *ruvA* coding sequence were used as a control. Cycle threshold values were calculated using the Lightcycler 480 software and converted to DNA concentrations based on a standard curve generated from 2-fold dilutions of *Caulobacter* genomic DNA. Fold enrichment values were calculated as ([P*_didA_*−output]/[*ruvA*−output])/([P*_didA_*−input]/[*ruvA*−input]). Error bars in Figure 7F were generated from technical triplicates, and the experiment shown is representative of biological duplicates.

## Supporting Information

Figure S1 **Cellular filamentation of** ***sidA*** **and** ***recA*** **mutants.** Wild-type, Δ*sidA*, and Δ*recA* cells were grown to mid-exponential in rich media and treated with 1 µg/ml MMC or left untreated. After 3 hours, cells were imaged by phase microscopy. Bar, 2 µm.
(TIF)

Figure S2 **Annotated gene expression profiles.** (A) Transcriptional profiles for the 50 most upregulated genes during DNA damage in wild-type cells (see Figure 1A) are shown with their corresponding CC numbers and NA1000 annotation. The “LexA” column shows genes whose upstream region contains a sequence match to 7 of the 8 bases in the *Caulobacter* LexA consensus binding site (GTTCN_7_GTTC) [15]. Genes whose log-fold changes post-damage in Δ*recA* cells are below 50% of those in wild-type cells are marked as “RecA-dependent.” All other genes are marked as “RecA-independent.” (B) The positions of microarray probes within CC3114 and CCNA03212 are shown below the genes as horizontal bars. The four right-most probes were used to calculate expression values for CCNA03212 (*didA*). (C) The transcriptional profiles for each probe in (B) are shown.
(TIF)

Figure S3 **DNA damage induction of DidA.** (A) Cells expressing *3×M2-didA* from the native, chromosomal P*_didA_* promoter were exposed to 1 or 3 µg/ml MMC or left untreated. Wild-type cells harboring a low- (pCT133) or medium- (pCT155) copy plasmid expressing *3×M2-didA* from P*_van_* were treated with or without vanillate. After 3 hours, cells were imaged by phase microscopy. Bar, 2 µm. (B) Samples from the experiments in (A) were taken at the times indicated and analyzed by Western blot using an α-FLAG/M2 antibody.
(TIF)

Figure S4 **DidA interacts with FtsN.** Bacterial two-hybrid analysis of interactions between T25-DidA (A) or T18-DidA (B) and cell division proteins fused to T18 or T25, respectively. Each pair was plated on LB, and colonies were restruck on MacConkey plates containing maltose.
(TIF)

Figure S5 **SidA interacts with FtsW.** (A) Cells expressing wild-type *ftsW* or *ftsW(A246T)* and overproducing M2-YFP-DidA for 2.5 hours were imaged by phase and epi-fluorescence microscopy. (B) Bacterial two-hybrid analysis of interactions between T18-M2-SidA and FtsW mutants fused to T25 as indicated. Colonies were grown to exponential phase in LB and 5 µl aliquots plated on MacConkey agar containing maltose.
(TIF)

Figure S6 **Suppressor mutant growth properties.** (A) Growth curves for the strains from Figure 5D grown in rich media. (B) Wild-type and *ftsW(A246T)* cells were grown to mid-exponential phase and imaged by phase microscopy. Cell lengths were quantified from 491 wild-type and 610 *ftsW(A246T)* cells using MicrobeTracker and summarized as a histogram with the maximum frequency for each strain normalized to 1. (C) Growth curves for wild-type, *ftsW(A246T)* and ftsW**I* cells grown in rich media. (D) Mixed populations of wild-type and *ftsW(A246T)* cells (*n*∼200) were imaged by time-lapse microscopy on PYE agarose pads. The times to first mid-cell division are shown. For raw data, see Data S3.
(TIF)

Figure S7 ***ftsW(A246T)*** **cells divide prematurely during MMC exposure.** Mixed populations of wild-type and *ftsW(A246T)* cells (*n*∼100) were imaged by time-lapse microscopy on PYE agarose pads containing 0.35 µg/ml MMC. The time to first mid-cell division and the percentage of cells that stopped growing following division are shown. Asterisks represent a statistically significant (*p*<0.01) difference relative to the wild type. Error bars represent standard error of the mean. For raw data, see Data S3.
(TIF)

Figure S8 **Induction at P** ***_sidA_*** **and P** ***_didA_*** **.** Wild-type cells harboring low-copy plasmids transcribing *egfp* from either P*_sidA_* or P*_didA_* were exposed to MMC (0.35 or 1.75 µg/ml) or cephalexin (5 or 35 µg/ml) for 1.5 or 3 hours. Samples were analyzed by Western blot with an α-EGFP antibody.
(TIF)

Figure S9 **Suppressors treated with cephalexin exhibit cell wall defects.** The strains from Figure 6D, grown to mid-exponential phase in rich media and treated with MMC or cephalexin for 6 hours and PI at 5 µM 1.5 hours before imaging. Cells were imaged by phase and fluorescence microscopy; representative populations are shown with PI+ cells false-colored red. Bar, 2 µm.
(TIF)

Figure S10 **Induction of** ***didA*** **during stress conditions.** Cells expressing *didA-3×M2* from the native, chromosomal *didA* promoter were treated with 3 µg/ml MMC, 1 and 3 mg/ml hydroxyurea (HU), 36 µg/ml cephalexin (ceph), and 10 and 100 µg/ml novobiocin (nov) for 1 hour each, ultraviolet light using a Stratalinker at energy setting 100 and 300 (UV), grown overnight in minimal medium (M2G), starved of glucose in minimal medium (- glu) for 30, 60, and 90 minutes, or treated with 5% and 10% ethanol (EtOH), 50 and 200 mM NaCl, 10 and 100 mM hydrogen peroxide (H_2_O_2_), 5 µg/ml kanamycin (kan), 1 µg/ml oxytetracycline (Tet), or 2 µg/ml chloramphenicol (chlor) for 45 minutes each. Samples were analyzed by Western blot using an α-FLAG/M2 antibody.
(TIF)

Table S1 **Strains, plasmids, and primers.**
(XLSX)

Data S1 **Microarray data for (A)** ***wt*** **and Δ** ***recA*** **cells treated with 1 µg/ml MMC for 30 minutes and (B)** ***wt*** **cells harboring pCT155:** ***P_van_-didA*** **treated with or without vanillate for 45 minutes.**
(XLSX)

Data S2 **Summary of growth and division defects following MMC treatment.**
(XLSX)

Data S3 **Raw data from** **Figures 6** **,** **7** **, S6, and S7.**
(XLSX)

Text S1 **Extended materials and methods.**
(DOCX)

## Abbreviations

ChIP: chromatin immunoprecipitation
MMC: mitomycin C
PI: propidium iodide
