# Supplementary figures and images for: A DNA Damage-Induced, SOS-Independent Checkpoint Regulates Cell Division in Caulobacter crescentus
Source: PLoS Biol. 2014 Oct 28;12(10):e1001977. doi: 10.1371/journal.pbio.1001977 (PMC4211646; doi:10.1371/journal.pbio.1001977)

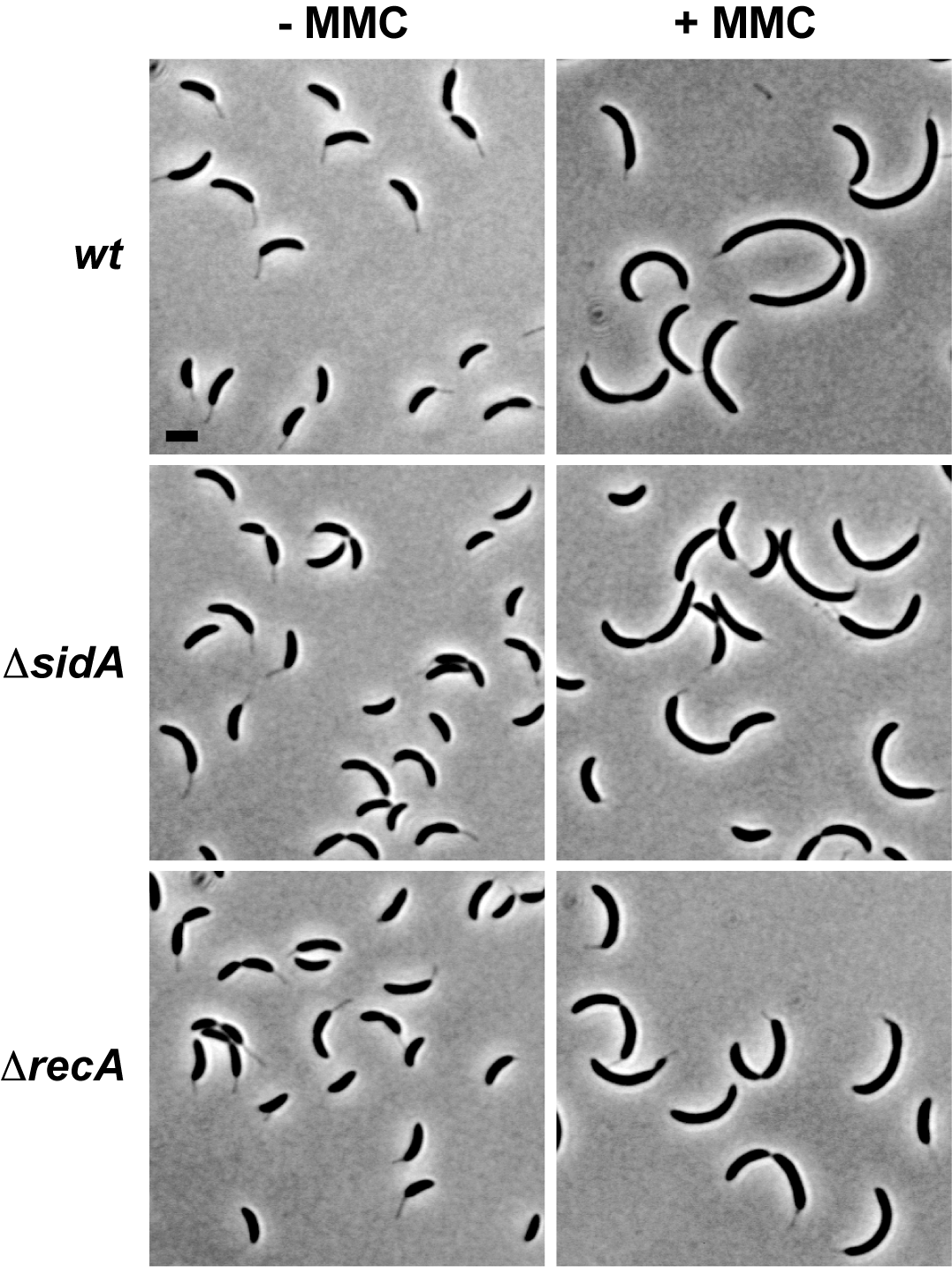

Supplement: Figure S1 — Cellular filamentation of sidA and recA mutants. Wild-type, ΔsidA, and ΔrecA cells were grown to mid-exponential in rich media and treated with 1 µg/ml MMC or left untreated. After 3 hours, cells were imaged by phase microscopy. Bar, 2 µm. (TIF) [file pbio.1001977.s001.tif]

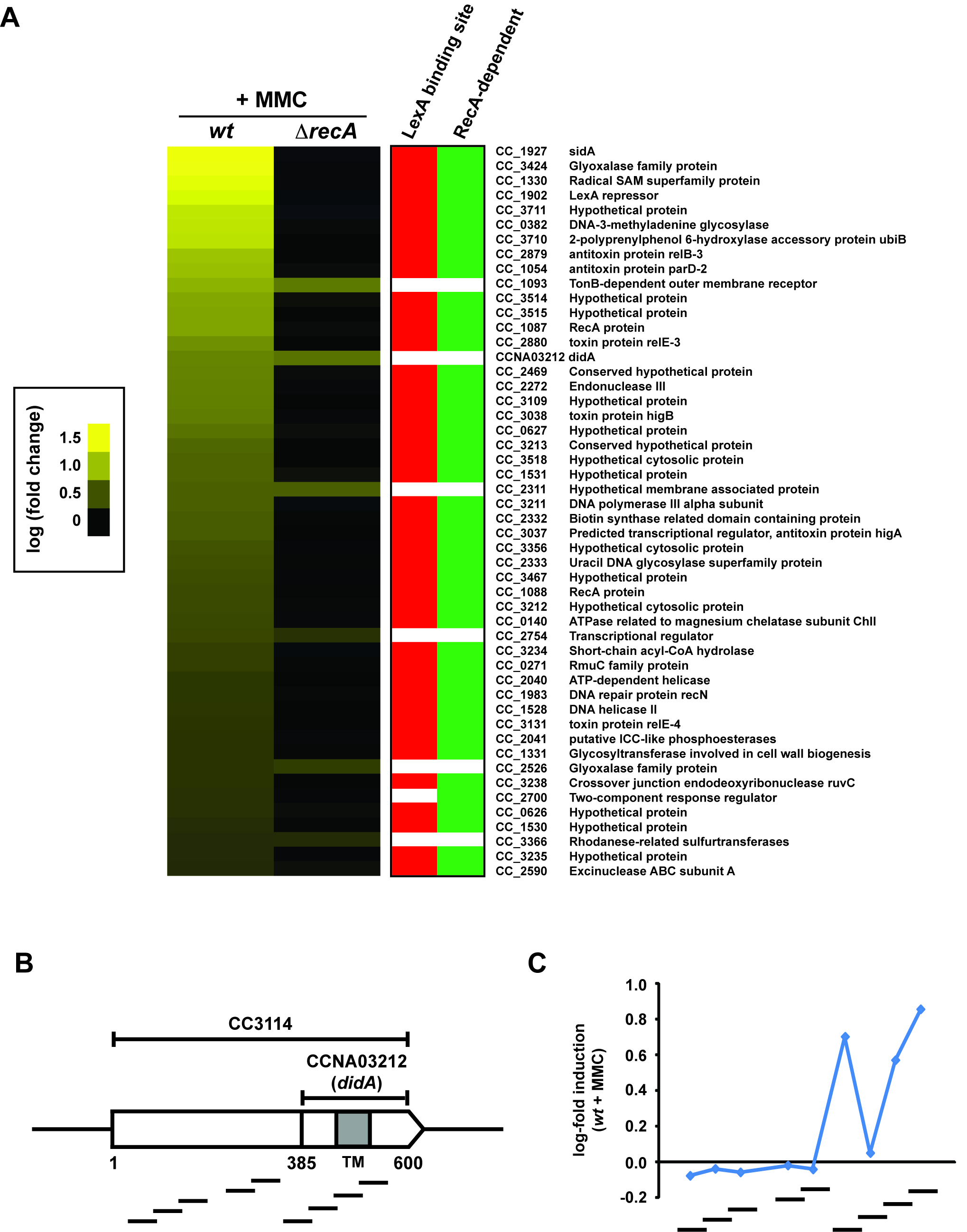

Supplement: Figure S2 — Annotated gene expression profiles. (A) Transcriptional profiles for the 50 most upregulated genes during DNA damage in wild-type cells (see Figure 1A) are shown with their corresponding CC numbers and NA1000 annotation. The “LexA” column shows genes whose upstream region contains a sequence match to 7 of the 8 bases in the Caulobacter LexA consensus binding site (GTTCN7GTTC) [15]. Genes whose log-fold changes post-damage in ΔrecA cells are below 50% of those in wild-type cells are marked as “RecA-dependent.” All other genes are marked as “RecA-independent.” (B) The positions of microarray probes within CC3114 and CCNA03212 are shown below the genes as horizontal bars. The four right-most probes were used to calculate expression values for CCNA03212 (didA). (C) The transcriptional profiles for each probe in (B) are shown. (TIF) [file pbio.1001977.s002.tif]

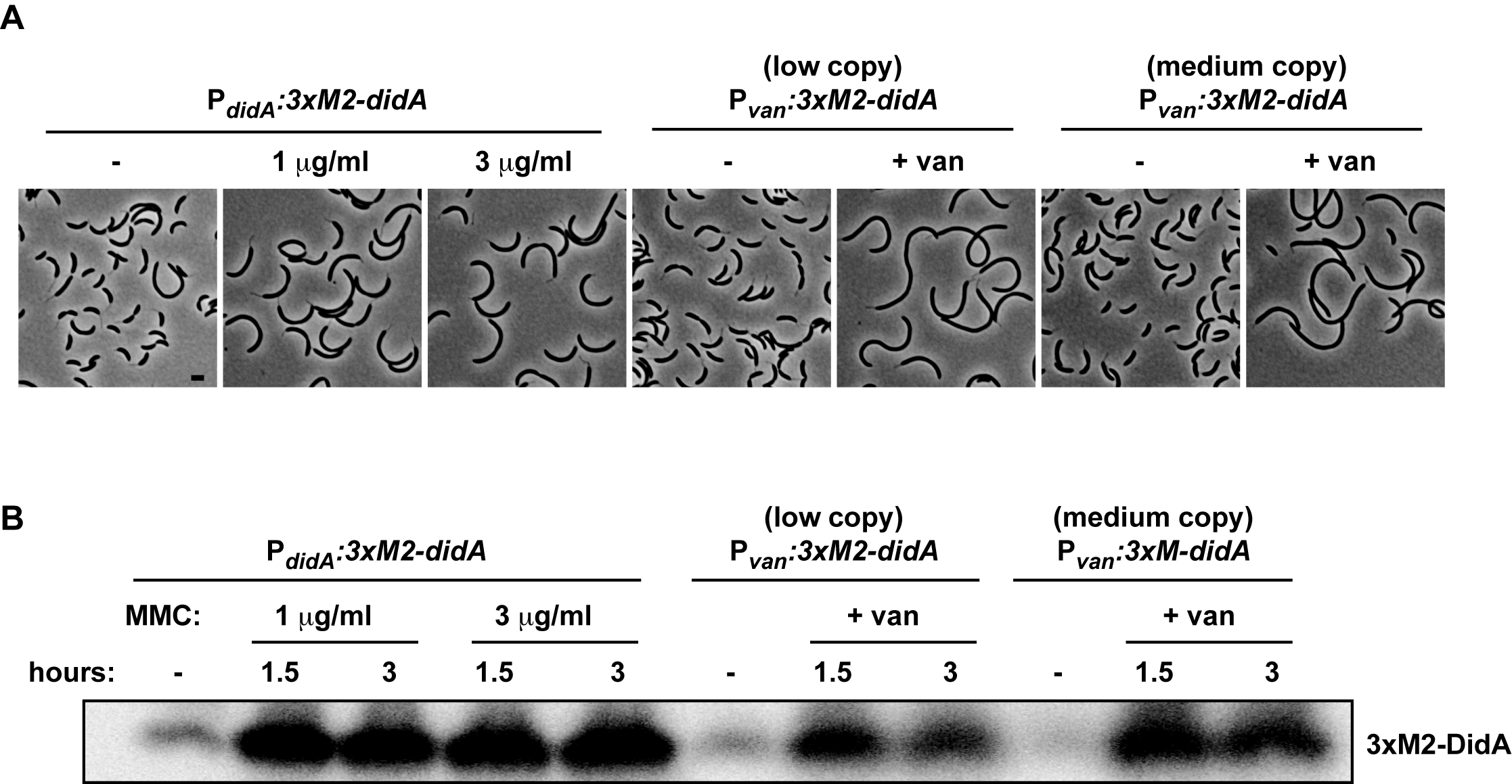

Supplement: Figure S3 — DNA damage induction of DidA. (A) Cells expressing 3×M2-didA from the native, chromosomal PdidA promoter were exposed to 1 or 3 µg/ml MMC or left untreated. Wild-type cells harboring a low- (pCT133) or medium- (pCT155) copy plasmid expressing 3×M2-didA from Pvan were treated with or without vanillate. After 3 hours, cells were imaged by phase microscopy. Bar, 2 µm. (B) Samples from the experiments in (A) were taken at the times indicated and analyzed by Western blot using an α-FLAG/M2 antibody. (TIF) [file pbio.1001977.s003.tif]

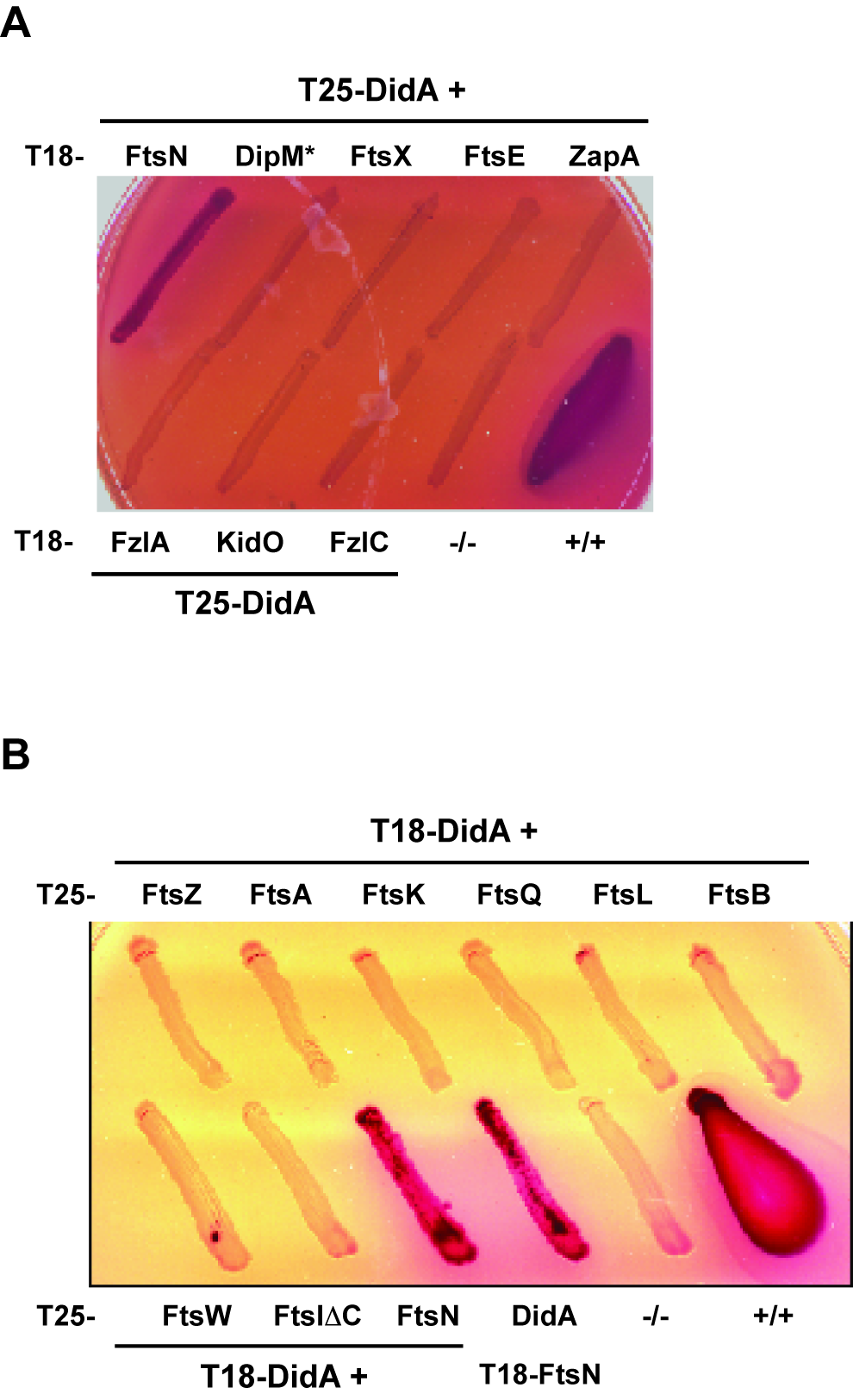

Supplement: Figure S4 — DidA interacts with FtsN. Bacterial two-hybrid analysis of interactions between T25-DidA (A) or T18-DidA (B) and cell division proteins fused to T18 or T25, respectively. Each pair was plated on LB, and colonies were restruck on MacConkey plates containing maltose. (TIF) [file pbio.1001977.s004.tif]

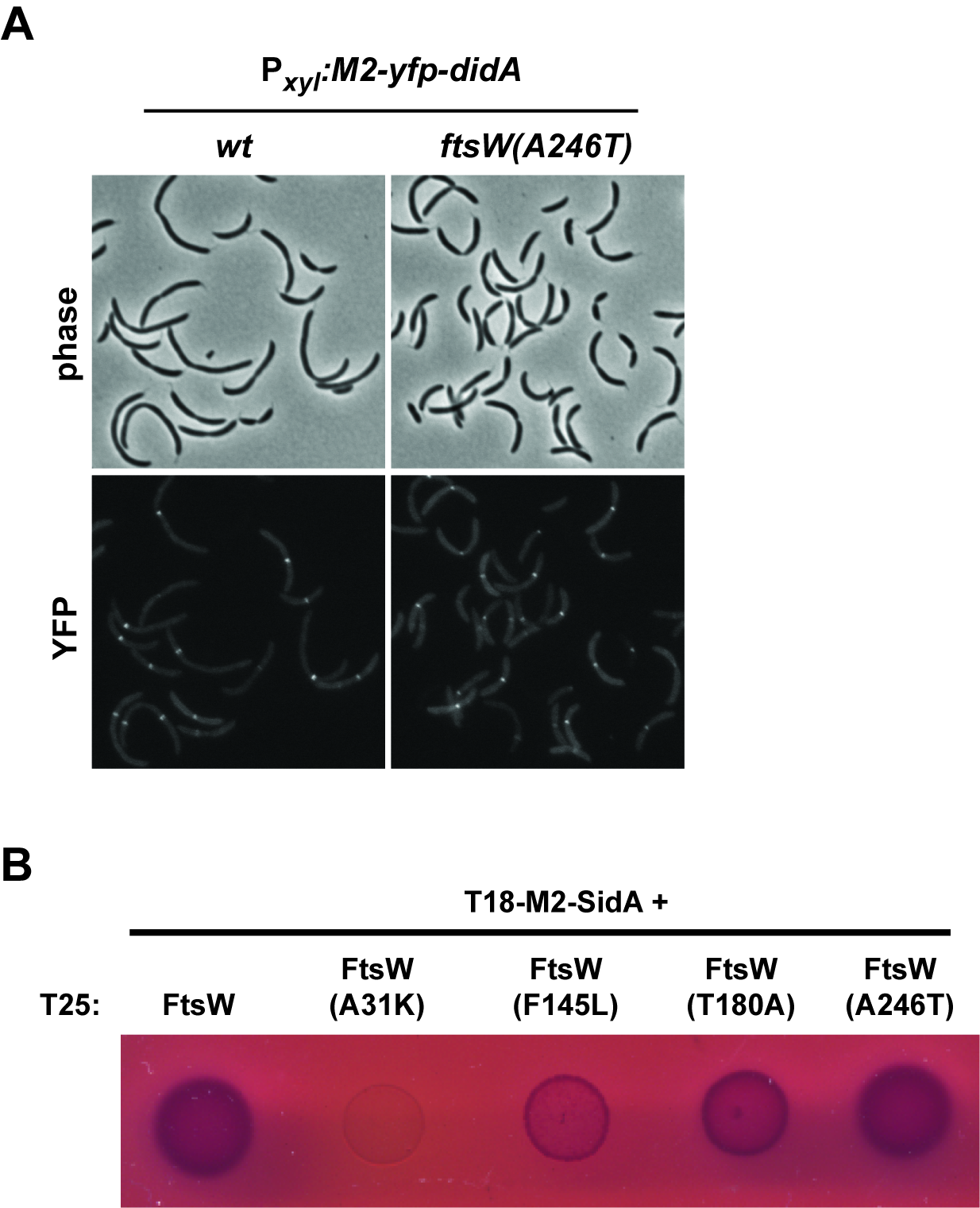

Supplement: Figure S5 — SidA interacts with FtsW. (A) Cells expressing wild-type ftsW or ftsW(A246T) and overproducing M2-YFP-DidA for 2.5 hours were imaged by phase and epi-fluorescence microscopy. (B) Bacterial two-hybrid analysis of interactions between T18-M2-SidA and FtsW mutants fused to T25 as indicated. Colonies were grown to exponential phase in LB and 5 µl aliquots plated on MacConkey agar containing maltose. (TIF) [file pbio.1001977.s005.tif]

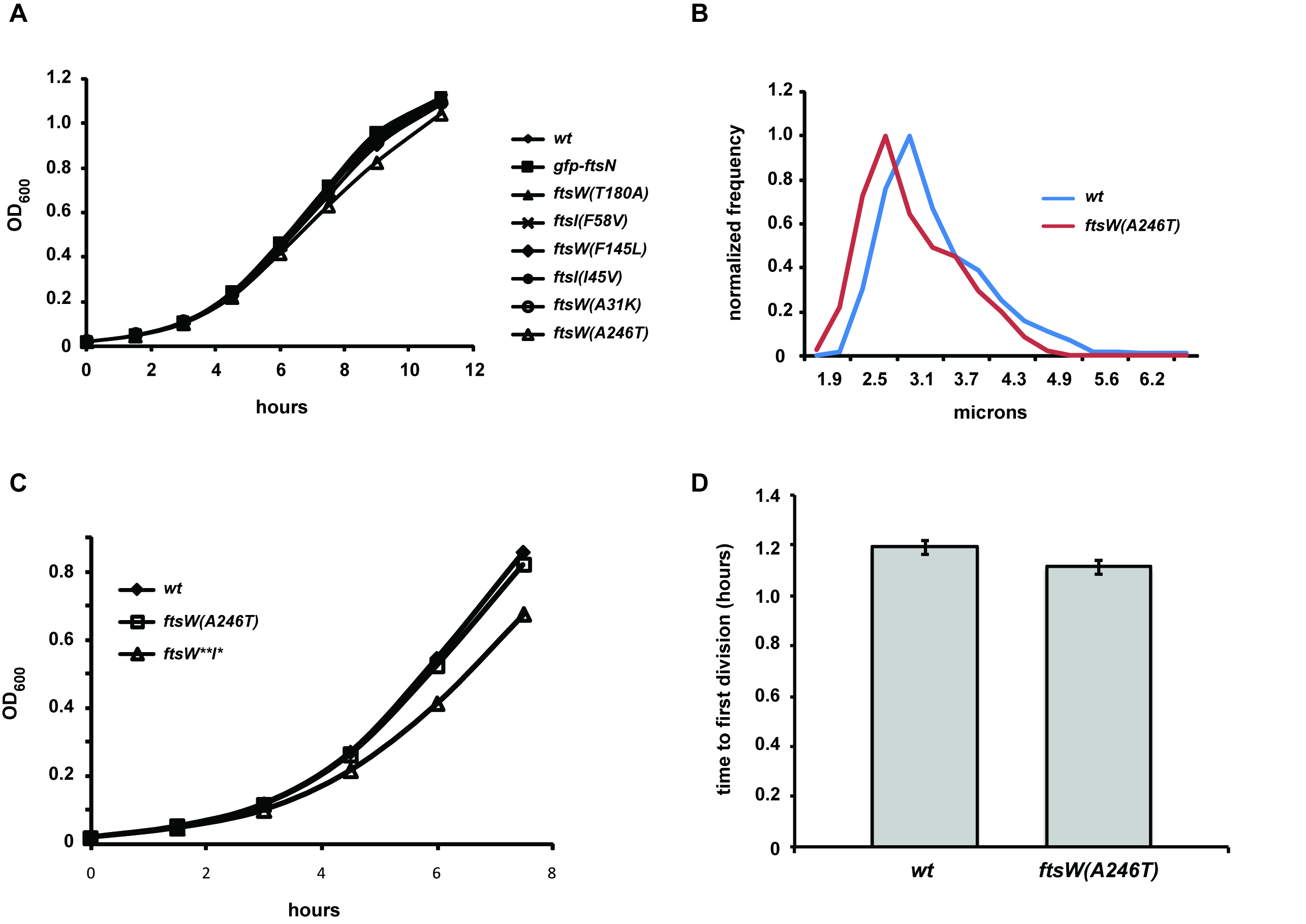

Supplement: Figure S6 — Suppressor mutant growth properties. (A) Growth curves for the strains from Figure 5D grown in rich media. (B) Wild-type and ftsW(A246T) cells were grown to mid-exponential phase and imaged by phase microscopy. Cell lengths were quantified from 491 wild-type and 610 ftsW(A246T) cells using MicrobeTracker and summarized as a histogram with the maximum frequency for each strain normalized to 1. (C) Growth curves for wild-type, ftsW(A246T) and ftsW**I* cells grown in rich media. (D) Mixed populations of wild-type and ftsW(A246T) cells (n∼200) were imaged by time-lapse microscopy on PYE agarose pads. The times to first mid-cell division are shown. For raw data, see Data S3. (TIF) [file pbio.1001977.s006.tif]

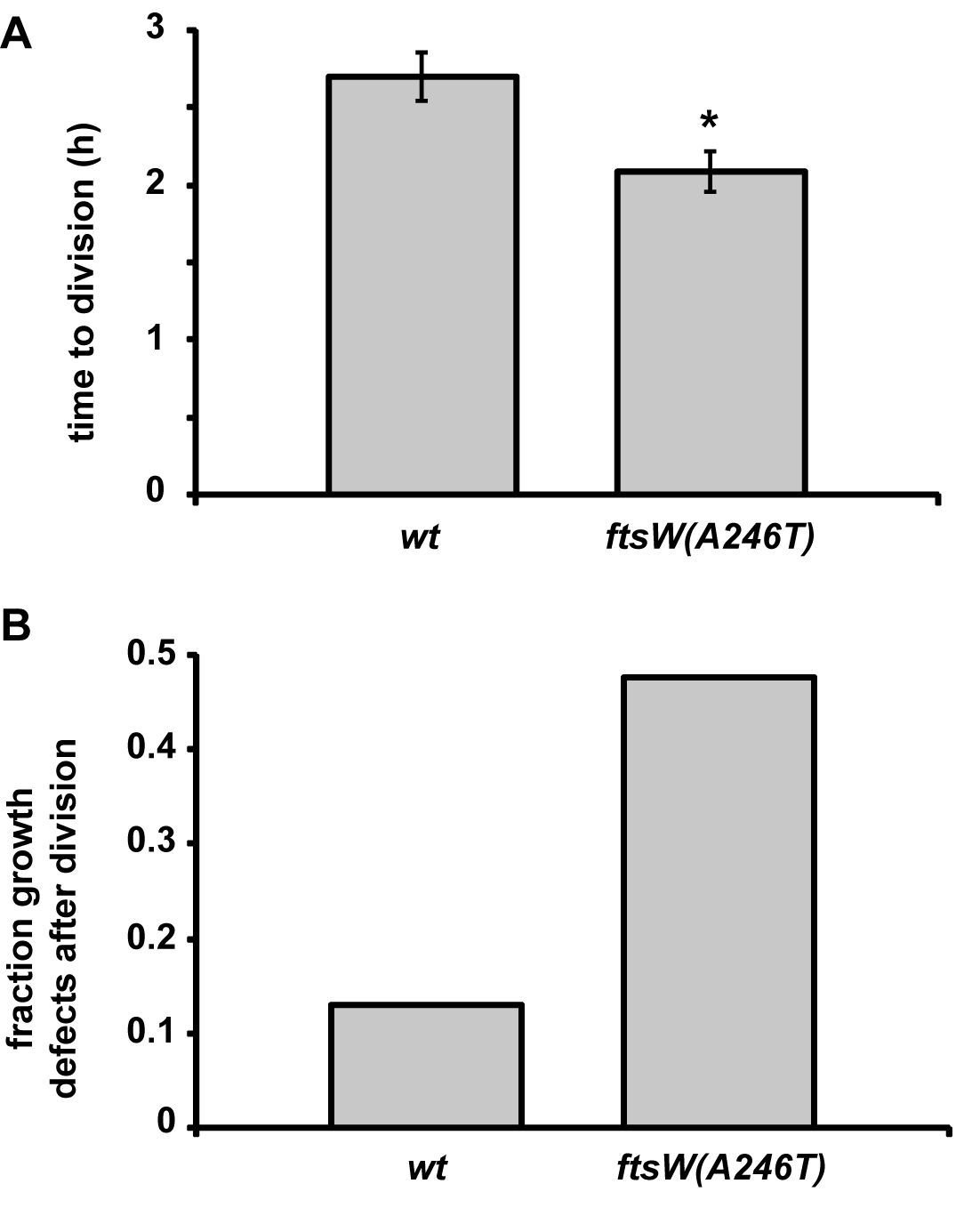

Supplement: Figure S7 — ftsW(A246T) cells divide prematurely during MMC exposure. Mixed populations of wild-type and ftsW(A246T) cells (n∼100) were imaged by time-lapse microscopy on PYE agarose pads containing 0.35 µg/ml MMC. The time to first mid-cell division and the percentage of cells that stopped growing following division are shown. Asterisks represent a statistically significant (p<0.01) difference relative to the wild type. Error bars represent standard error of the mean. For raw data, see Data S3. (TIF) [file pbio.1001977.s007.tif]

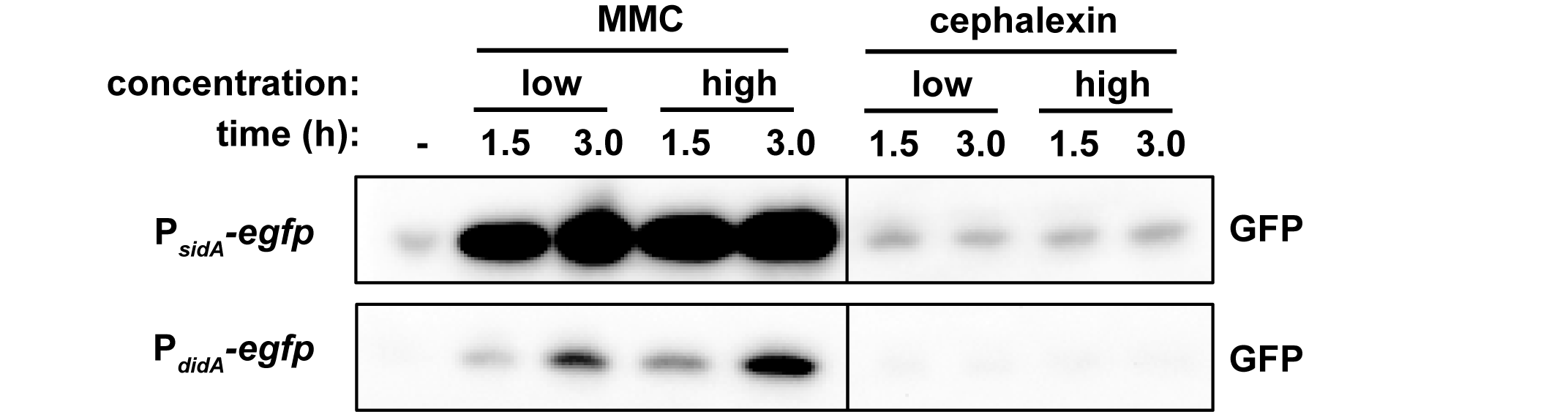

Supplement: Figure S8 — Induction at P sidA and P didA . Wild-type cells harboring low-copy plasmids transcribing egfp from either PsidA or PdidA were exposed to MMC (0.35 or 1.75 µg/ml) or cephalexin (5 or 35 µg/ml) for 1.5 or 3 hours. Samples were analyzed by Western blot with an α-EGFP antibody. (TIF) [file pbio.1001977.s008.tif]

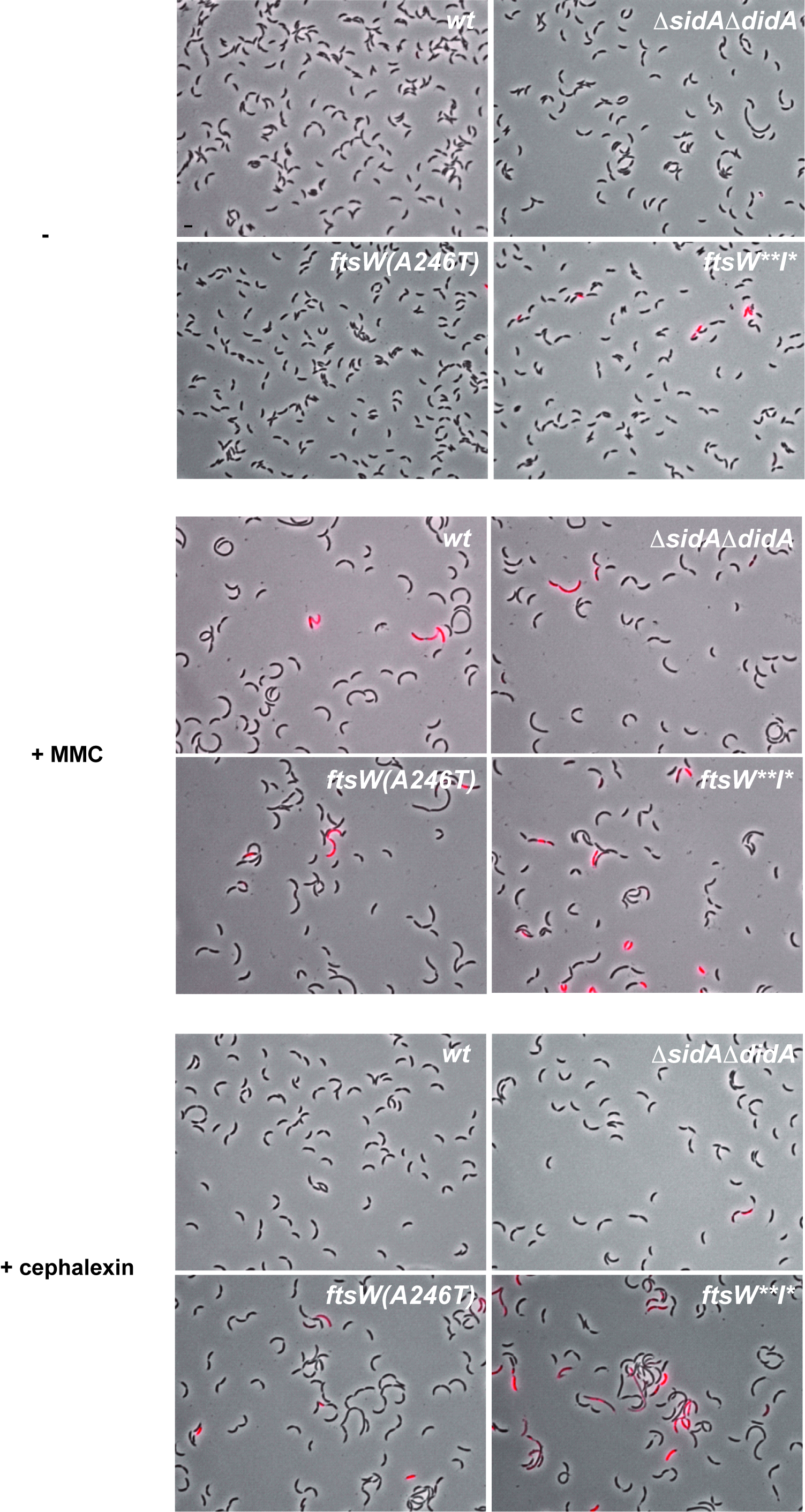

Supplement: Figure S9 — Suppressors treated with cephalexin exhibit cell wall defects. The strains from Figure 6D, grown to mid-exponential phase in rich media and treated with MMC or cephalexin for 6 hours and PI at 5 µM 1.5 hours before imaging. Cells were imaged by phase and fluorescence microscopy; representative populations are shown with PI+ cells false-colored red. Bar, 2 µm. (TIF) [file pbio.1001977.s009.tif]

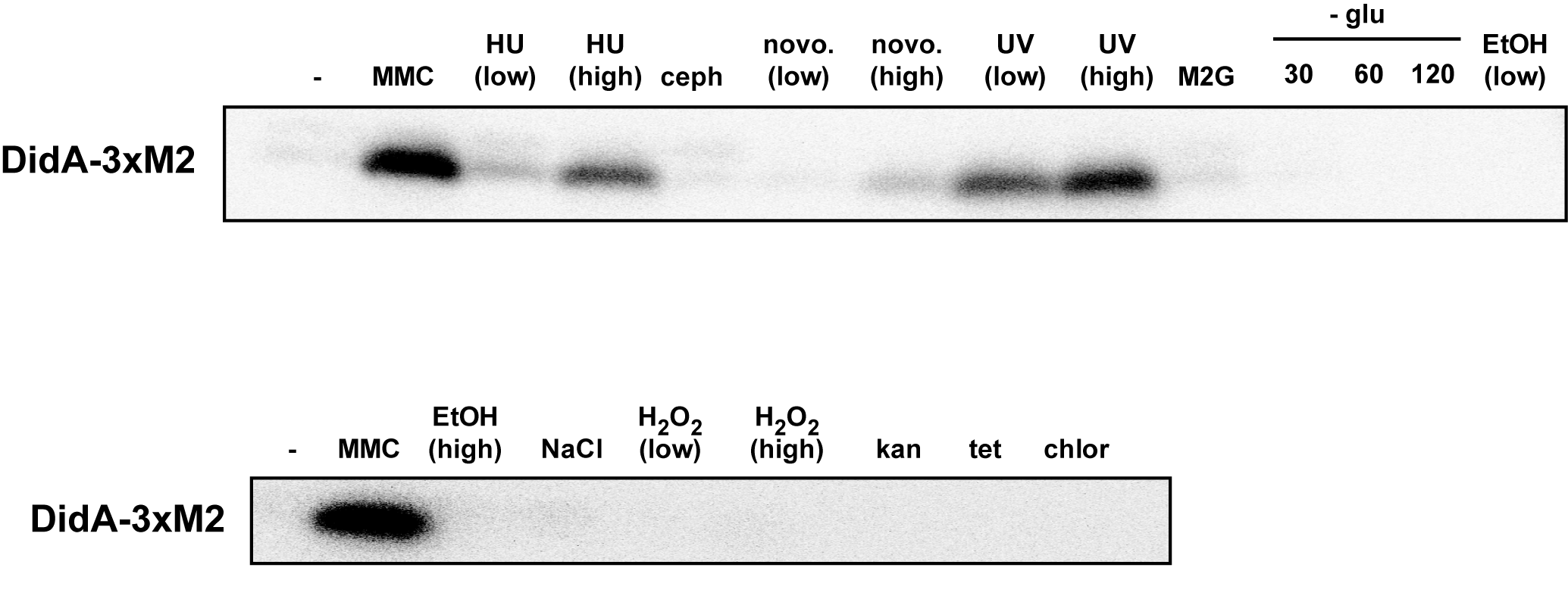

Supplement: Figure S10 — Induction of didA during stress conditions. Cells expressing didA-3×M2 from the native, chromosomal didA promoter were treated with 3 µg/ml MMC, 1 and 3 mg/ml hydroxyurea (HU), 36 µg/ml cephalexin (ceph), and 10 and 100 µg/ml novobiocin (nov) for 1 hour each, ultraviolet light using a Stratalinker at energy setting 100 and 300 (UV), grown overnight in minimal medium (M2G), starved of glucose in minimal medium (- glu) for 30, 60, and 90 minutes, or treated with 5% and 10% ethanol (EtOH), 50 and 200 mM NaCl, 10 and 100 mM hydrogen peroxide (H2O2), 5 µg/ml kanamycin (kan), 1 µg/ml oxytetracycline (Tet), or 2 µg/ml chloramphenicol (chlor) for 45 minutes each. Samples were analyzed by Western blot using an α-FLAG/M2 antibody. (TIF) [file pbio.1001977.s010.tif]
