# Supplementary material for: A DNA Damage-Induced, SOS-Independent Checkpoint Regulates Cell Division in Caulobacter crescentus
Source: PLoS Biol. 2014 Oct 28;12(10):e1001977. doi: 10.1371/journal.pbio.1001977 (PMC4211646; doi:10.1371/journal.pbio.1001977)
Supplement: Text S1 — Extended materials and methods. (DOCX) [file pbio.1001977.s015.docx]

# Text S1: Extended Materials and Methods

## Growth conditions

Caulobacter strains were grown at 30°C in rich medium (PYE). To induce or repress expression from P*_xyl_*, liquid and plated media were supplemented with 0.3% xylose or 0.2% glucose, respectively. To induce or repress expression from P_van_, liquid and plated media were supplemented with or without 500 µM vanillic acid (Fluka). Mitomycin C (A&G) was added to liquid cultures at 1 µg/ml and to agarose pads and agar plates at 0.35 µg/ml unless otherwise indicated. To induce expression from P_lac_, plate media were supplemented with 75 or 100 µM IPTG (Sigma). Novobiocin (Fluka) and cephalexin (Sigma) were added to agar plates at 0.35 µg/ml and 7.5 µg/ml respectively. Antibiotics were used at the following concentrations for strain construction and plasmid maintenance in *Caulobacter* cells: kanamycin, 5 μg/ml in liquid and 25 μg/ml in plates; oxytetracycline, 1 μg/ml in liquid and 2 μg/ml in plates; chloramphenicol, 2 μg/ml in liquid and 1 μg/ml in plates; gentamycin, 2.5 µg/ml in liquid and 5 µg/ml in plates. Antibiotics were used at the following concentrations for BTH101 *E. coli* cells in MacConkey agar plates: carbenicillin, 100 µg/ml; kanamycin, 50 µg/ml; streptinomycin, 100 µg/ml. Transformations and transductions were performed as previously described (Ely, 1991).

## Plate dilutions

For plate dilution assays, cells were grown to stationary phase and diluted 10-fold six times yielding approximate ODs of 1.0, 0.1, 0.01, 0.001, 0.0001 and 0.00001. 5 µl of each dilution was spotted onto agarose plates with different dilutions along the x axis and different strains along the y axis.

## Strain and plasmid construction

*Plasmid construction*

All PCR amplifications were performed with *Caulobacter* CB15N genomic DNA unless otherwise noted. 3xM2 fragments were amplified from mini-prepped plasmids containing the *3xM2* sequence.

To create pNPT-spec-*P_didA_-didA-3xM2*, a fragment spanning the upstream homology region of *didA* to just before the stop codon was amplified with the primers *didA_UHR_F* and *didA_UHR_R*, the 3xM2 epitope was amplified with *3xM2_F_+didAUHR* and *3xM2_R_TAG* and a fragment containing the region immediately downstream of the *didA* coding sequence was amplified with *didA_DHR_F_+3xM2* and *didA_DHR_R*. The three fragments were fused by a fusion PCR with the primers *didA_UHR_F* and *didA_DHR_R*, and cloned into pNPT-spec-DEST at the EcoRI restriction sites.

To create pNPT-spec-P*_didA_-3xM2-didA*, a fragment containing the upstream homology region of *didA* was amplified with the primers *didA_UHR_F* and *PdidA_R*, a fragment containing *3xM2* was amplified with the primers *3xM2_F_+PdidA* and *3xM2_R_noStop*, and a fragment containing *didA* and the downstream homology region was amplified with the primers *did_F_+3xM2* and *didA_DHR_R*. The three fragments were fused by a fusion PCR with the primers *didA_UHR_F* and *didA_DHR_R*, and cloned into pNPT-spec-DEST at the EcoRI restriction sites.

To create pNPT-spec-*ftsN* region, *ftsN* and its upstream and downstream homology regions were amplified with the primers *ftsN_UHR_F* and *ftsN_DHR_R2*. This fragment was cloned into pNPT-spec-DEST at the EcoRI restriction sites.

To create pNPT-spec-*ftsN(L202P)*, pNPT-spec-*ftsN(P156S)* and pNPT-spec-*ftsN(F252L)*, mutations were introduced by site-directed mutagenesis of pNPT-spec-*ftsN* region with the primers *ftsN(T605C)_F* and *ftsN(T605C)_R*, *ftsN(C466T)_F* and *ftsN(C466T)_R*, or *ftsN(T754C)_F* and *ftsN(T754C)_R* respectively.

To create pNPT-spec-*lexA(K203A)*, a fragment containing the region upstream from lexA resiude K203 was amplified with the primers *lexA _UHR_F* and *lexA(K203A)_UHR_R*, and a downstream fragment was amplified with *lexA(K203A)_DHR_F* and *lexA_DHR_R*. The K203A mutation was introduced in the overhang region of each primer. The two fragments were fused by a fusion PCR with the primers *lexA _UHR_F* and *lexA_DHR_R* and cloned into pNPT-spec-DEST at the EcoRI restriction sites.

To create pNPT-spec-P*_ftsW_-venus-ftsW*, a fragment spanning the upstream regulatory region of *ftsW* to just before the start codon was amplified with the primers *PftsW_UHR_F* and *PftsW_UHR_R*, a fragment containing the coding sequence of *venus* was amplified from pVVENN-2 with *Venus_F_+PftsW* and *Venus_R*, and a fragment containing the coding sequence of *ftsW* was amplified with *PftsW_DHR_F_+Venus* and *PftsW_DHR_R*. The three fragments were fused by a fusion PCR with the primers *PftsW_UHR_F* and *PftsW_DHR_R* and cloned into pNPT-spec-DEST at the EcoRI restriction sites.

To create pNPT-spec*-ftsW(A246T)*, a fragment containing the region surrounding *ftsW* residue A246 was amplified with the primers *ftsW_region_F* and *ftsW_region_R* and cloned into pNPT-spec-DEST at the EcoRI restriction site. The *ftsW(A246T)* mutation was introduced by a site-directed mutagenesis PCR reaction with the primers *ftsW(A246T)_F* and *ftsW(A246T)_R*.

To create pNPT-spec-*ftsW(F145L,A246T)*, the *ftsW(A246T)* mutation was introduced by a site-directed mutagenesis PCR reaction into pNPT-spec-*ftsW(F145L)* with the primers *ftsW(A246T)_F* and *ftsW(A246T)_R*.

To create pNPT-spec-*ΔdidA*, a fragment spanning the upstream region of *didA* through the first three codons was amplified with the primers *didA_UHR_F* and *didA_UHR_R* and a fragment spanning the last three codons through the downstream region was amplified with *didA_DHR_F_+UHR* and *didA_DHR_R*. The two fragments were fused by a fusion PCR with the primers *didA_UHR_F* and *didA_DHR_R* and cloned into pNPT-spec-DEST at the EcoRI restriction sites.

To create pNPT-spec-*hfaB-P_didA_-lacZ-hfaB*, a fragment containing an hfaB upstream homology region was amplified with the primers *hfaB_UHR_F* and *hfaB_UHR_R*, a fragment containing the *didA* promoter was amplified with the primers *PdidA_F_+hfaB* and *PdidA_R*, a fragment containing *lacZ* was amplified from *E. coli* MG1655 genomic DNA with the primers *lacZ_F_+PdidA* and *lacZ_R_+hfaB*, and a fragment containing an hfaB downstream homology region was amplified with the primers *hfaB_DHR_F* and *hfaB_DHR_R*. These fragments were fused by a fusion PCR with the primers *hfaB_UHR_F* and *hfaB_DHR_R* and cloned into pNPT-spec-DEST at the SpeI and AflII restriction sites.

To create pNPT-spec-*ΔdriD*, a fragment containing the first three amino acids of *driD* and the upstream homology region were amplified with the primers *driD_UHR_F* and *driD_UHR_R*, and a fragment containing the last ten amino acids of *driD* and the downstream homology region were amplified with the primers *driD_DHR_F_+UHR* and *driD_DHR_R*. These fragments were fused by a fusion PCR with the primers *driD_UHR_F* and *driD_DHR_R* and cloned into pNPT-spec-DEST at the EcoRI restriction sites.

To create pCT133-P*_didA_-didA-3xM2*, a fragment containing the *didA* promoter and coding sequence fused at its C-terminus to the 3xM2 epitope was amplified from the strain ML2088 with the primers *PdidA_F_CACC* and *didA_DHR_R*. This fragment was cloned into pENTR and transferred by an LR reaction into pCT133.

To create pCT133-P*_van_-didA* and pCT155-P*_van_-didA*, a fragment containing the *vanA* promoter was amplified with the primers *Pvan+r_F_CACC* and *Pvan_R* and a fragment containing the *didA* conding sequence was amplified with *didA_F_+Pvan* and *didA_R*. The two fragments were fused by a fusion PCR with the primers *Pvan+r_F_CACC* and *didA_R*, cloned into pENTR and transferred by LR reactions into either pCT133 or pCT155.

To create pCT133-P*_sidA_-sidA*, a fragment containing the *sidA* promoter and coding sequence was amplified with the primers *PsidA_F* and *sidA_R*, cloned into pENTR and transferred by an LR reaction into pCT133.

To create pCT133-P*_didA_-didA*, a fragment containing the *didA* promoter and coding sequence was amplified with the primers *PdidA_F* and *didA_R*, cloned into pENTR and transferred by an LR reaction into pCT133.

To create pCT133-P*_lac_-3xM2-didA*, a fragment containing the P*_lac_* promoter was amplified with the primers *Plac_F_SLIC* and *Plac_R_SLIC* from *E. coli* MG1655 genomic DNA, and a fragment containing *3xM2-didA* was amplified with the primers *3xM2_F_SLIC* and *DidA_R_SLIC* from pCT155-P*_van_-3xM2-didA*. These fragments were cloned by SLIC (Li and Elledge, 2012) into pCA19, which is the pENTR vector with an insertion of a 100 bp MCS from pRXMCS-2 (Aakre CD, (Thanbichler et al., 2007)).

To create pCT133-P*_didA_-egfp*, a fragment containing P*_didA_* was amplified with the primers *PdidA_F_CACC* and *PdidA_R* and a fragment containing *egfp* was amplified with the primers *egfp_F_+PdidA* and *egfp_R* from pCT133-P*_sidA_-egfp*. The fragments were fused by a fusion PCR with the primers *PdidA_F_CACC* and *egfp_R*, cloned into pENTR and transferred by an LR reaction to pCT133.

To create pCT133-P*_xyl_-ftsW-egfp*, a fragment containing P*_xyl_* was amplified with the primers *Pxyl_F_CACC* and *Pxyl_R*, and a fragment containing *ftsW-egfp* was amplified with the primers *ftsW_F_+Pxyl* and *egfp_R* from the pGFPC-2 plasmid containing a C-terminal GFP fusion to *ftsW*. The fragments were fused by a fusion PCR with the primers *Pxyl_F_CACC* and *egfp_R,* cloned into pENTR and transferred by an LR reaction to pCT133.

To create pCT133-P*_driD_-driD*, a fragment containing P*_driD_-driD* was amplified with the primers *PdriD_F_CACC*  and *driD_R*. This fragment was cloned into pENTR and transferred by an LR reaction to pCT133.

To create pCT133-P*_driD_-3xM2-driD*, a fragment containing P*_driD_* was amplified with the primers *PdriD_F_CACC* and *PdriD_R*, a fragment containing *3xM2* was amplified with the primers *3xM2_F_+PdriD* and *3xM2_R_noStop*, and a fragment containing *driD* was amplified with the primers *driD_F_+3xM2* and *driD_R*. The fragments were fused by a fusion PCR with the primers *PdriD_F_CACC* and *driD_R,* cloned into pENTR and transferred by an LR reaction to pCT133.

To create pCT133-P*_driD_-driD-3xM2*, a fragment containing P*_driD_–driD* was amplified with the primers *PdriD_F_CACC* and *driD_R_noTAG*, and a fragment containing *3xM2* was amplified with the primers *3xM2_F_+driD* and *3xM2_R*. The fragments were fused by a fusion PCR with the primers *PdriD_F_CACC* and *3xM2_R,* cloned into pENTR and transferred by an LR reaction to pCT133.

To create pML498-*yfp-didA*, a fragment containing the coding sequence of *yfp* was amplified with the primers *yfp_F* and *yfp_R*, and a fragment containing the coding sequence of *didA* was amplified with *didA_F_+yfp* and *didA_R*. The two fragments were fused by a fusion PCR with the primers *yfp_F* and *didA_R*, cloned into pENTR and transferred by an LR reaction to pML498.

To create pCT133-P*_van_-3xM2-didA* and pCT155-P*_van_-3xM2-didA*, a fragment containing the *vanA* promoter was amplified with the primers *Pvan+r_F_CACC* and *Pvan_R*, a fragment containing the 3xM2 epitope was amplified from a 3xM2 template with *3xM2_F_+Pvan* and *3xM2_R_noStop*, and a fragment containing the coding sequence of *didA* was amplified with *did_F_+3xM2* and *didA_R*. The three fragments were fused by a fusion PCR with the primers *Pvan+r_F_CACC* and *didA_R*, cloned into pENTR and transferred by an LR reaction to pCT133 and pCT155 respectively.

To create pBXMCS-2-*ftsN***, a mutant library of *ftsN* was amplified with the primers *ftsN_F_ndeI* and *ftsN_b2h_R_ecoRI* using the PCR conditions described in the Materials and Methods section and cloned into pBXMCS-2 using the NdeI and EcoRI restriction sites.

To create bacterial two-hybrid (BACTH) plasmids, fragments containing *Caulobacter* cell division genes were amplified using the primers indicated in the ‘BACTH primers’ section of Tabls S2 and cloned into pKT25 or pUT18C at the KpnI and EcoRI restriction sites creating N-terminal fusions with several exceptions.

The *Caulobacter* *ftsA* gene was fused to the transmembrane domain of *E. coli malF* and cloned into pKT25 or pUT18C at the BamHI and EcoRI restriction sites. Specifically, a fragment containing the *ftsA* coding sequence minus the last 15 amino acids was amplified with the primers *ftsA_b2h_F_bamHI* and *ftsA_b2h_R* and a fragment containing the *malF* transmembrane domain was amplified from MG1655 with the primers *malF-TM_soe_ftsA_F* and *malF-TM_R_ecoRI*. The two fragments were fused by a fusion PCR with the primers *ftsA_b2h_F_bamHI* and *malF-TM_R_ecoRI*.

The coding regions of *ftsK* and *kidO* were cloned into pKNT25 and pUT18 using the HindIII and XbaI restriction sites creating C-terminal fusions. The periplasmic protein DipM was fused to the MalG transmembrane domain as described (Möll et al., 2010).

To create pUT18C-*malF-ftsN*, a fragment containing the transmembrane domain of *malF* was amplified with the primers *malF_b2h_F_kpnI* and *malF_b2h_R*, and a fragment containing the periplasmic domains of *ftsN* was amplified with the primers *ftsN_b2h_F_+malF* and *ftsN_b2h_R_ecoRI*. The fragments were fused by fusion PCR with the primers *malF_b2h_F_kpnI* and *ftsN_b2h_R_ecoRI* and cloned into pUT18C using the KpnI and EcoRI sites.

To create pUT18C-*ftsNΔC* and pUT18C-*ftsNΔLC*, fragments of *ftsN* lacking either the C-terminal SPOR domain or the periplasmic linker and SPOR domains were amplified with the primers *ftsN_b2h_F_kpnI* and *ftsN_b2h_deltaC_R_ecoRI* or *ftsN_b2h_deltaLC_R_ecoRI* respectively. Each fragment was cloned into pUT18C at the KpnI and EcoRI sites.

To create pUT18C-*ftsN(H1)*, *ftsN* with its periplasmic helix 1 (H1) replaced with an unstructured sequence from SpmX was amplified from the strain AM80 (Möll and Thanbichler, 2009) with the primers *ftsN_b2h_F_kpnI* and *ftsN_b2h_R_ecoRI*.

To create pUT18C-*ftsN(L202P)*, pUT18C-*ftsN(P156S)* and pUT18C-*ftsN(F252L)*, site-directed mutagenesis was performed on the template pUT18C-*ftsN* with the primers *ftsN(T605C)_F* and *ftsN(T605C)_R*, *ftsN(C466T)_F* and *ftsN(C466T)_R*, or *ftsN(T754C)_F* and *ftsN(T754C)_R* respectively.

*Strain construction*

To generate ML2088, ML2091, ML2097, ML2103, ML2111, ML2148, ML2150, ML2156, ML2157, ML2158 and ML2174, the inserts in plasmids pNPT-spec-P*_didA_-didA-3xM2*, pNPT-spec-*lexA(K203A)*, pNPT-spec-P*_ftsW_-venus-ftsW*, pNPT-spec*-ftsW(A246T)*, pNPT-spec-*ΔdidA*, pNPT-spec-*P_didA_-3xM2-didA*, pNPT-spec-*P_ftsN_-3xM2-ftsN*, pNPT-spec-*ftsN(L202P)*, pNPT-spec-*ftsN(P156S)*, pNPT-spec-*ftsN(F252L)* and pNPT-spec-*ΔdriD* respectively were introduced into the wild-type CB15N genome by two-step recombination.

To generate ML 2112, the insert in plasmid pNPT-spec-*ΔdidA* was introduced into ML1759 by two-step recombination.

To generate ML2159, the insert in plasmid pNPT-spec-*ftsW(F145L,A246T)* was introduced into ML1885 by two-step recombination.

To generate ML2180 and ML2181, the insert in plasmid pNPT-spec-*ΔdriD* was introduced into ML1759 and ML21088 respectively by two-step recombination.

To generate ML2089, ML2090 and ML2092, the plasmid pCT133-P*_didA_-didA-3xM2* was introduced into CB15N, ML743 and ML2091 respectively by electroporation.

To generate ML2093, ML2094, ML2095 and ML2149 the plasmids pCT133-P*_van_-didA*, pCT155-P*_van_-didA*, pML498-*yfp-didA* and pCT133-*P_van_-3xM2-didA* respectively were introduced into CB15N by electroporation.

To generate ML2151 and ML2152, the plasmids pML396-*P_xyl_-egfp-sidA* and pML396-*P_xyl_-eyfp-didA* were introduced into ML2150 by electroporation.

To generate ML2096 and ML2102, the plasmid pCT155-P*_van_-3xM2-didA* was introduced into ML1681 and CB15N respectively by electroporation.

To generate ML2098, ML2099, ML2100, and ML2101, the plasmid pCT155-P*_van_-didA* was introduced into MT196, ML2097, CJW2144 and MT46 respectively by electroporation.

To generate ML2104, ML2160, ML2161 and ML2162, the plasmid pML1716-*sidA* was introduced into ML2103, ML2156, ML2157 and ML2158 by electroporation.

To generate ML2105, ML2106, ML2107, ML2108, ML2109 and ML2110, the plasmid pCT155-P*_van_-didA* was introduced into ML1884, ML1771, ML1772, ML2103, ML1885 and ML1773 by electroporation.

To generate ML2113 and ML 2114, the plasmids pCT133-P*_sidA_-sidA* and pCT133-P*_didA_-didA* respectively were introduced into ML2112 by electroporation.

To generate ML2154, ML2163, ML2164, ML2165, ML2166, ML2167 and ML2169, pCT133-*P_lac_-3xM2-didA* was introduced into AM52, CB15N, MT46, ML2103, ML2156, ML2157 and ML2158 by electroporation.

To generate ML2155, the mutant *ftsN* library in pBXMCS-2 was frozen down after transformation of *E. coli* Top10 cells with the pBXMCS-2-*ftsN*** ligation.

To generate ML2169, the insert in plasmid pNPT-spec-*hfaB-P_didA_-lacZ-hfaB* was introduced into ML2111 by two-step recombination.

To generate ML2175 and ML2176, the plasmid pCT133-P*_sidA_-egfp* was introduced into ML2174 and ML743 by electroporation.

To generate ML2177, ML2178 and ML2179, the plasmid pCT133-P*_didA_-egfp* was introduced into CB15N, ML2174 and ML743 by electroporation.

To generate ML2182, ML2183, ML2184 and ML2185, the plasmids pCT133-*P_xyl_-ftsW-egfp*, pCT133-*P_driD_-driD*, pCT133-*P_driD_-3xM2-driD* and pCT133-*P_driD_-driD-3xM2* were introduced into ML2181 by electroporation.

To generate ML2186 and ML2187, the plasmids pCT133-P*_driD_-driD* and pCT133-P*_driD_-driD-3xM2* were introduced into ML2174 by electroporation.

## Image analysis and time-lapse microscopy

To determine cell lengths, phase images were segmented using the MATLAB-based software package MicrobeTracker (Sliusarenko et al., 2011). For time-lapse microscopy, cells were immobilized on 1.5% agarose pads made with PYE medium and supplemented with 0.35 µg/ml MMC where indicated. Cells were imaged for 8 hours in a glass-bottomed petri dish wrapped with Parafilm and maintained at 30°C using the Zeiss Temp Module S1 and Heating Insert P S1. Image capture and processing was done through Metamorph software (Universal Imaging Group). Automatic focusing was performed using the Zeiss Definite Focus module and images were taken every 15 minutes. The ‘time to first division’ was calculated by visual inspection in a double-blind fashion such that the scorer did not know which strain was being analyzed. Specifically, the first frame when the two new daughter cell poles were visibly disengaged was recorded. Additionally, ‘growth defects following divisions’ were recorded if both daughter cells ceased growth/elongation following a mid-cell division event.

To determine cell envelope integrity, cells were stained with 5 μM propidium iodide for 1.5 hours prior to imaging. Fluorescent images taken in the mCherry/RFP channel were normalized in imageJ to minimum and maximum displayed values of 104 and 292 respectively. Cells appearing red were scored as PI+ cells with compromised envelope integrity.

## PCR conditions

PCR was performed with Phusion HF DNA polymerase using 5X Phusion GC Reaction Buffer (NEB). Each reaction contained 10 µl buffer, 10 µl 3M Betaine monohydrate (Sigma), 4 µl dNTPs, 5 of a 10 µM forward and reverse primer mix, 50 ng template, 1 µl DMSO, 0.5 µl polymerase and nuclease-free water to 50 µl. Two-step cycling was performed as follows: 98°C 30 sec, 34x(98°C 10 sec, 72°C 30 sec/kb), 72°C 5 min. Fusion PCR was performed similarly with 50 ng of the largest template fragment and equimolar amounts of the smaller template fragments and an additional annealing step of 20 s at 60°C was added. Site-directed mutagenesis PCR was performed as described (Chen et al., 2009).

## Two-step recombination

Allelic replacements and chromosomal deletions were performed by a two-step recombination protocol as described (Skerker et al., 2005).

## Gateway cloning

Gateway cloning (Invitrogen) was performed as described (Skerker et al., 2005).

## ChIP analysis

Cells were treated with sodium phosphate (pH 7.6) and formaldehyde at final concentrations of 10 mM and 1% respectively and incubated for 10 minutes at room temperature. The reaction was quenched with 100 mM glycine and incubated for 5 minutes at room temperature and then 15 minutes on ice. Cells were pelleted at 8500 rpm at 4°C and washed 3x in ice-cold 1X PBS (pH 7.4). After the final spin, pellets were resuspended in 500ul TES buffer (10 mM Tris-HCl (pH 7.5), 1 mM EDTA, 100 mM NaCl) supplemented with 35,000 U of Ready-Lyse (Epicentre) and

incubated for 15 minutes at room-temperature. To the lysate, 500 µl of ChIP buffer (1.1% Triton X-100, 1.2 mM EDTA, 16.7 mM Tris-HCl (pH 8.1), 167 mM NaCl) supplemented with cOmplete protease inhibitor tablets (Roche) was added and samples were incubated at 37°C for 10 minutes. Samples were sonicated on ice and centrifuged at 14,000 rpm for 5 minutes at 4°C. The supernatant was normalized to 0.5 mg/ml in ChIP buffer (+ 0.01% SDS) and pre-cleared with 50 µl of Protein-A dynabeads (Invitrogen) that had been preblocked overnight with 100 µg BSA in 1 ml ChIP buffer. After rotating for 1 hour at 4°C, the beads were pelleted and 10% of the supernatant was saved as "input" chromatin and frozen at -80°C. To the remaining supernatant, 5 µl of α-Flag antibody was added followed by rotation overnight at 4°C. The immune complexes were captured with 50 µl pre-blocked Protein-A dynabeads by rotation for 2 hours at 4C. The beads were recovered and washed by rotation for 15 minutes at 4°C in the following buffers: (1) Low salt wash buffer (0.1% SDS, 1% Triton X-100, 2mM EDTA, 20 mM Tris-HCl (pH 8.1), 150 mM NaCl); (2) High salt wash buffer (0.1% SDS, 1% Triton X-100, 2mM EDTA, 20 mM Tris-HCl (pH 8.1), 500 mM NaCl); (3) LiCl wash buffer (0.25 M LiCl, 1% NP-40, 1% deoxycholate, 1mM EDTA, 10mM Tris-HCl (pH 8.1); (4) Twice with TE buffer (10 mM Tris-HCl (pH 8.1) and 1 mM EDTA). The complexes were eluted twice by adding fresh elution buffer (1% SDS, 0.1 M NaHCO3), vortexing, and rotating at RT for 15 minutes. In parallel, the input DNA was brought to 500 µl in elution buffer. To the input and output eluates, 37.5 µl 4M NaCl and 1 µg RNase A were added and the samples were incubated at 65°C overnight. Samples were supplemented with 40 mM EDTA and 40 mM Tris-HCl (pH 6.5), and 100 µg Proteinase K (NEB) were added, followed by incubation at 45°C for 2 hours. DNA was extracted with phenol:chloroform:isoamyl alcohol and resuspended in TE buffer.

# References (for Text S1)

Chen, Y. E., C. G. Tsokos, E. G. Biondi, B. S. Perchuk, and M. T. Laub, 2009, Dynamics of two phosphorelays controlling cell cycle progression in *Caulobacter* *crescentus*: J Bacteriol, v. 191, p. 7417-29.

Ely, B., 1991, Genetics of *Caulobacter* *crescentus*: Methods Enzymol, v. 204, p. 372-84.

Li, M. Z., and S. J. Elledge, 2012, SLIC: a method for sequence- and ligation-independent cloning: Methods Mol Biol, v. 852, p. 51-9.

Möll, A., S. Schlimpert, A. Briegel, G. J. Jensen, and M. Thanbichler, 2010, DipM, a new factor required for peptidoglycan remodelling during cell division in *Caulobacter* *crescentus*: Mol Microbiol, v. 77, p. 90-107.

Möll, A., and M. Thanbichler, 2009, FtsN-like proteins are conserved components of the cell division machinery in proteobacteria: Mol Microbiol, v. 72, p. 1037-53.

Skerker, J. M., M. S. Prasol, B. S. Perchuk, E. G. Biondi, and M. T. Laub, 2005, Two-component signal transduction pathways regulating growth and cell cycle progression in a bacterium: a system-level analysis: PLoS Biol, v. 3, p. e334.

Sliusarenko, O., J. Heinritz, T. Emonet, and C. Jacobs-Wagner, 2011, High-throughput, subpixel precision analysis of bacterial morphogenesis and intracellular spatio-temporal dynamics: Mol Microbiol, v. 80, p. 612-27.

Thanbichler, M., A. A. Iniesta, and L. Shapiro, 2007, A comprehensive set of plasmids for vanillate- and xylose-inducible gene expression in *Caulobacter* *crescentus*: Nucleic Acids Res, v. 35, p. e137.
